# CaMKK2 Regulates Macrophage Polarization Induced by Matrix Stiffness: Implications for Shaping the Immune Response in Stiffened Tissues

**DOI:** 10.1002/advs.202417778

**Published:** 2025-03-04

**Authors:** Ya Guan, Min Zhang, Jiyeon Song, Marcos Negrete, Tyler Adcock, Reeva Kandel, Luigi Racioppi, Sharon Gerecht

**Affiliations:** ^1^ Department of Biomedical Engineering Duke University Durham NC 27708 USA; ^2^ Division of Hematological Malignancies and Cellular Therapy Department of Medicine Duke University Medical Center Durham NC 27708 USA; ^3^ Department of Molecular Medicine and Medical Biotechnology University of Naples Federico II Naples Italy

**Keywords:** hydrogels, macrophages, mechanics, pro‐regenerative, stiffness

## Abstract

Macrophages are essential for immune responses and maintaining tissue homeostasis, exhibiting a wide range of phenotypes depending on their microenvironment. The extracellular matrix (ECM) is a vital component that provides structural support and organization to tissues, with matrix stiffness acting as a key regulator of macrophage behavior. Using physiologically relevant 3D stiffening hydrogel models, it is found that increased matrix stiffness alone promoted macrophage polarization toward a pro‐regenerative phenotype, mimicking the effect of interleukin‐4(IL‐4) in softer matrices. Blocking Calcium/calmodulin‐dependent kinase kinase 2 (CaMKK2) selectively inhibited stiffness‐induced macrophage polarization without affecting IL‐4‐driven pro‐regenerative pathways. In functional studies, CaMKK2 deletion prevented M2‐like/pro‐tumoral polarization caused by matrix stiffening, which in turn hindered tumor growth. In a murine wound healing model, loss of CaMKK2 impaired matrix stiffness‐mediated macrophage accumulation, ultimately disrupting vascularization. These findings highlight the critical role of CaMKK2 in the macrophage mechanosensitive fate determination and gene expression program, positioning this kinase as a promising therapeutic target to selectively modulate macrophage responses in pathologically stiff tissues.

## Graphical abstract

Macrophage phenotypes are influenced by the microenvironment. Matrix stiffness, a key ECM factor, polarizes macrophage toward a pro‐regenerative phenotype in a 3D physiological environment. CaMKK2 selectively regulates stiffness‐induced macrophage polarization, impacting tumor growth and wound healing. This study underscores CaMKK2's vital role in macrophage mechanosensitive fate, making it a potential therapeutic target for modulating macrophage responses in stiff pathological tissues.

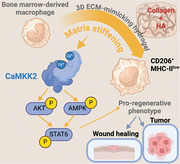

## 1 Introduction

Macrophages are highly plastic cells that are critical innate and adaptive immunity mediators. In response to a changing environment, macrophages polarize toward a spectrum of phenotypes, enabling a plethora of biological functions in development, disease, and regeneration.^[^ ^1^, ^2^, ^3^, ^4^, ^5^ ^]^ Macrophages have been conventionally classified into M1 and M2 phenotypes.^[^ ^6^, ^7^ ^]^ However, it is now recognized that this strict categorization fails to capture the full complexity of macrophage phenotypes.^[^ ^8^ ^]^ Macrophages in different tissues and microenvironments may express a combination of M1 and M2 markers, suggesting a more nuanced and dynamic activation state.^[^ ^9^, ^10^ ^]^

Macrophage fate is strongly determined by the surrounding extracellular matrix (ECM). ECM biomechanical properties, such as spatial confinement, stiffness, and viscoelasticity, have been shown to play essential roles in modulating macrophage phenotypes.^[^ ^11^, ^12^, ^13^, ^14^ ^]^ Among these ECM cues, matrix stiffening is a significant characteristic in many diseases and has been shown to regulate macrophage fate in various studies.^[^ ^15^, ^16^, ^17^, ^18^, ^19^, ^20^, ^21^, ^22^, ^23^ ^]^ Substrates with varying stiffness, on which cells are cultured, have been used in to investigate how ECM stiffness directs macrophage polarization. Nonetheless, using a 3D matrix is desired to more accurately recapitulate the macrophage niche in the physiological environment. In addition, many studies used neoplastic cell lines such as RAW 264.7,^[^ ^16^, ^18^ ^]^ THP‐1,^[^ ^17^ ^]^ and U937^20^, which exhibit the typical macrophage phenotype but are less ideal for modeling the macrophages derived from bone marrow progenitor cells and blood monocytes. As a result, studies in this field have reported inconsistent or even contradictory outcomes.^[^ ^15^, ^16^, ^17^, ^18^, ^19^, ^20^, ^21^ ^]^

Understanding how ECM stiffness guides and modulates macrophage fate has important physio‐pathological implications, which can lead to the identification of new druggable targets and effective countermeasures. Previous studies have identified various mechanosensors through which macrophages sense the changes in substrate stiffness, and such might be expanded to 3D. For example, changes in ECM stiffness can be captured by integrins and translated into activation of the Hippo pathway by triggering YAP/TAZ activation.^[^ ^24^, ^25^ ^]^ Mechanical forces can alter the tension in the plasma membrane of cells, forcing mechanosensitive ion channels to open. In macrophages, Piezo1 and TRPV4 are two essential ion channels, and the opening of these channels allows the entry of extracellular Ca^2+^ ions.^[^ ^22^, ^26^ ^]^ The influx of Ca^2+^ ions can activate various downstream pathways and has a profound impact on macrophage fate.^[^ ^27^, ^28^ ^]^ Calcium/calmodulin‐dependent kinase kinase 2 (CaMKK2) is a protein kinase, that is activated when the intracellular Ca^2+^ level rises.^[^ ^29^ ^]^ We originally identified CaMKK2 as a key regulator in granulocyte development and macrophage activation programs.^[^ ^30^, ^31^ ^]^ More recently, we demonstrated that deletion of *Camkk2* in myeloid cells stimulates the anti‐tumor immune response, pointing out this protein as a critical druggable target to reshape the immunosuppressive tumor microenvironment (TME).^[^ ^32^, ^33^, ^34^ ^]^ However, the molecular mechanisms linking CaMKK2 to key macrophage functions remain largely undefined. Given the involvement of this kinase in cytoskeletal remodeling, we were prompted to investigate the role of CaMKK2 in the activation of the macrophage mechanosensing program.

We develop a collagen‐based hydrogel to culture primary bone marrow‐derived macrophages (BMDM) in 3D. In this system, we can induce hydrogel crosslinking “on‐demand,” providing a dynamically stiffened environment for the macrophages. We tackle the limitations of existing methods to isolate BMDM, which frequently lead to low cell yields and adversely affect the activation programs and functional responses of the recovered BMDM.^[^ ^35^ ^]^ Integrating advanced culture technique of BMDM and a 3D hydrogel with the capacity to stiffen into our in vitro system and complementary tumor and wound healing mouse models, we test the hypothesis that an increase in matrix stiffness can modulate macrophage fate and that the phenotypic polarization further shapes their cellular responses and orchestrates immune activities.

## 2 Results

### 2.1 “On‐Demand” Stiffening Hydrogels as ECM Mimics for Macrophages

**Figure 1:**
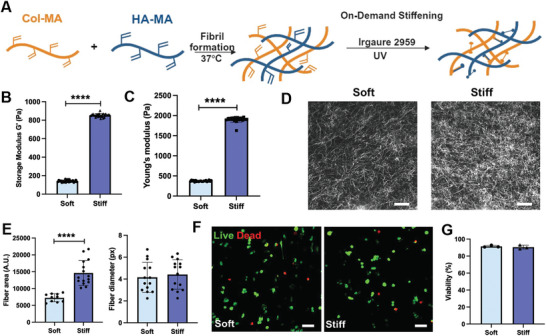
Fabrication and characterizations of the dynamically stiffening hydrogel. A) Schematic of the fabrication of collagen‐hyaluronic acid (Col‐HA) hydrogels to encapsulate BMDMs. B) Rheology measurement of the storage modulus of the hydrogels. N = 3. (*t*‐test, *p*<0.0001) C) AFM measurement of the Young's modulus of Col‐HA hydrogel. N = 3 with at least 20 measurements in each gel; (*t*‐test, *p*<0.0001) D) Representative reflective confocal images of the fiber structure of Col‐HA hydrogel. Scale bar = 20 µm. E) Analysis of fiber area and fiber diameter. N = 3 with at least 10 images in each gel; (*t*‐test, fiber area *p*<0.0001; fiber diameter p = 0.62) F) Representative live/dead staining images of BMDMs encapsulated in the hydrogels for 24 h. Scale bar = 100 µm. G) Quantification of the cell viability (N = 3; *t*‐test p = 0.58).

Monocyte‐derived macrophages adapt to specific cues and signals in their microenvironment to effectively carry out their immune functions. To study macrophage fate decisions we used BMDM. Common methods for harvesting macrophages grown on regular tissue culture‐treated polystyrene surfaces include mechanical scraping, treatment with calcium chelators like EDTA, and enzymatic methods such as trypsin. These methods frequently lead to low cell yield because of strong adhesion, notable effects on activation programs, and the functional response of recovered BMDM.^[^ ^35^ ^]^ Further, cell populations showing less ability to adhere and higher resistance to mechanical and chemical stress associated with detachment are probably positively selected using this traditional approach. To minimize these confounding effects, we generated BMDM using ultra low‐attachemnt plastic, as reported previously.^[^ ^30^ ^]^ Cells generated using this strategy maintain a high percentage of viability, preserving their monocyte‐like nature before 3D encapsulation. To study ECM stiffness, one of the essential physical cues in the macrophage niche, we utilized a dynamically stiffened hydrogel system modified from our published model (**Figure** **1**A).^[^ ^36^ ^]^ The hydrogel is based on collagen and hyaluronic acid (HA). Collagen is a major component of the ECM that provides structural support to tissues and maintains tissue integrity.^[^ ^37^ ^]^ HA is another key component of the ECM and provides membrane receptors, such as CD44, to facilitate macrophage adhesion.^[^ ^38^, ^39^ ^]^ Both components were modified with a methacrylate group to induce chemical cross‐linking.

We optimized the biomaterial platform for matrix concentration and ultraviolet (UV) exposure time (**Table** **1**) to generate hydrogels with different stiffnesses. We used shear rheology and atomic force microscopy (AFM) to measure the storage modulus (G’, Figure 1B) and Young's modulus (E, Figure 1C; Figure S1A, Supporting Information), respectively. The hydrogels were classified as soft or stiff based on their storage and Young's moduli values. The soft hydrogels had storage and Young's moduli values of 100 and 370 Pa, respectively. The stiff hydrogels had storage and Young's moduli values of 840 and 1900 Pa, respectively. We confirmed that such UV exposure time would not affect macrophage viability or phenotype (Figure S1B,C, Supporting Information). We also used higher collagen concentration for stiff gel (6 mg mL^−1^) than soft gel (4 mg mL^−1^). We proved that hydrogel with higher collagen concentration but without chemical crosslinking did not show a significant increase in stiffness (by only 25 Pa, Figure S1D, Supporting Information), indicating that the stiffening from soft to stiff gel is primarily attributed to stronger crosslinking, not matrix protein concentration.

**Table 1:** Chemical constituents and crosslinking conditions of the soft and stiff Col‐HA hydrogels.

	Col‐MA conc.	HA‐MA conc.	Irgacure 2959 conc.	UV light exposure
Soft	4 mg mL^−1^	1 mg mL^−1^	/	/
Stiff	6 mg mL^−1^	1 mg mL^−1^	2.5 mg mL^−1^	300 s

It has been reported that collagen I crosslinking leads to changes in fiber density and structure.^[^ ^40^ ^]^ Here, we used reflective confocal imaging to analyze the fibers. The fiber density was substantially increased in the stiff group, compared with soft gel (Figure 1D), with a significantly increased fiber coverage area (Figure 1E). However, no difference was observed in fiber diameter between different conditions, indicating that matrix stiffening was caused primarily by higher crosslink density, not by changes in the intrinsic collagen/HA fiber structure. Finally, we also evaluated whether matrix stiffening affects cell viability. BMDM were mixed with gel precursors incubated at 37 °C to allow fibril formation. After the cells adhered and interacted with the matrix, we induced UV crosslinking “on demand” to enhance the crosslinking density and dynamically increase the stiffness. The live/dead imaging demonstrates that BMDMs maintain similar viability between both conditions (Figure 1F,G). Around 90% of viability is commonly observed for a 3D cell culture in hydrogels.^[^ ^41^ ^]^ Thus, the dynamically stiffened hydrogel system offers a distinctive opportunity to embed the macrophages in a 3D matrix and then controllably modify stiffness to examine their responses.

### 2.2 Macrophage's Phenotypic Polarization Upon Dynamic Stiffening

We next sought to investigate whether matrix stiffening modulates macrophage fate. Previous studies have shown that substrate stiffness (2D culture) regulates macrophage fate, altering their morphology, phenotypic, and transcriptional changes.^[^ ^15^, ^16^, ^17^, ^18^, ^19^, ^20^, ^21^, ^22^, ^23^ ^]^ Here, we first focus on the impact of matrix stiffness (3D culture) on macrophage fate.

**Figure 2:**
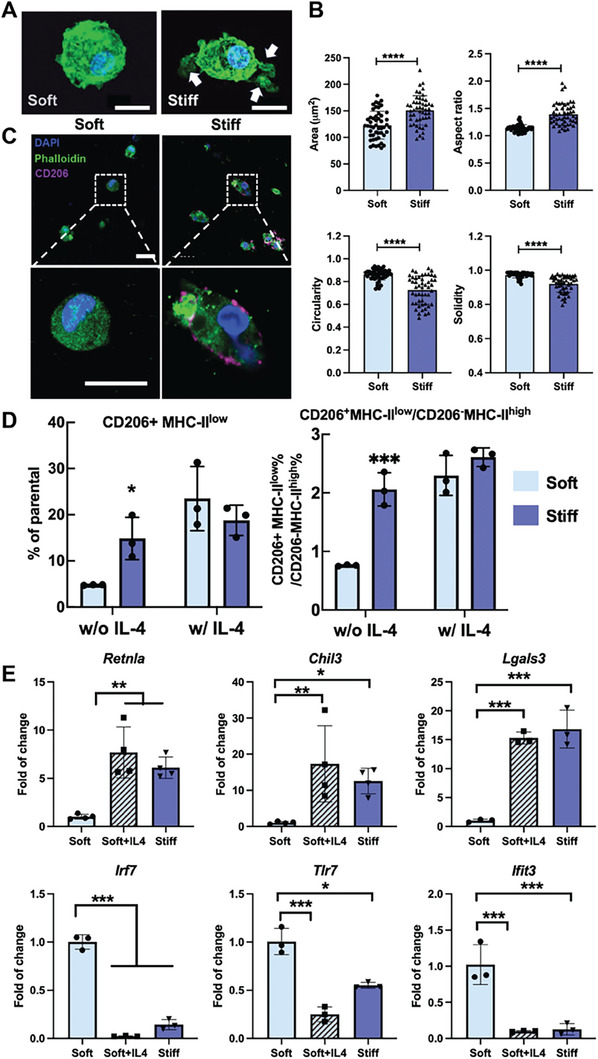
BMDM's phenotypic polarization upon dynamic stiffening. A) Representative confocal images of BMDMs (green: phalloidin; blue: DAPI) encapsulated in the hydrogels for 2 days. Scale bar = 20 µm. Maximum intensity projections of Z‐stack images were used. B) Quantification of the size, elongation, circularity, and solidity of BMDMs. N = 3 with 50 measurements in each group; (*t*‐test, *p*<0.0001 for size, elongation, circularity, and solidity) C) Representative confocal images of BMDMs stained with CD206 (magenta), DAPI (blue), and phalloidin (green). Scale bar = 20 µm. Maximum intensity projections of Z‐stack images were used. D) Flow cytometry analysis of CD206 and MHC‐II expression on CD11b^+^F4/80^+^ gated BMDMs encapsulated in the hydrogels after 2 days. The percentage of two subpopulations (CD206^+^MHC‐II^low^ and CD206^−^MHC‐II^high^) and their ratio was calculated (t‐test, *p<0.05, ***p<0.001). E) RT‐qPCR analysis of expression of pro‐regenerative genes (*Retnla*, *Chil3*, and *Lgals3*) and pro‐inflammatory genes (*Irf7*, *Tlr7*, and *Ifit3*) in BMDMs in soft, stiff, and soft+IL‐4 hydrogels (One‐way ANOVA, *p<0.05, **p<0.01, ***p<0.001).

Macrophage morphology is closely related to their phenotype. Unpolarized M0 macrophages appear as round cells; pro‐inflammatory (or conventionally known as M1 macrophages) are larger and flattened with extended pseudopods; pro‐regenerative (also named M2‐like macrophages) are elongated and granular.^[^ ^42^ ^]^ Thus, we first examined the effect of stiffness on the morphology of macrophages. No significant morphological changes could be detected when culturing BMDMs in the soft gel, while matrix stiffening significantly affected their form. We found that when the stiffness increased, BMDMs changed their morphology from a rounded or amoeboid shape to a more elongated or spindle‐like shape (**Figure** **2**A). Quantification further validated that BMDMs had an increased size and elongation, and a decreased circularity and solidity when ECM stiffness rises (Figure 2B). Protrusion formation is also associated with macrophage polarization.^[^ ^43^ ^]^ More protrusions (Figure 2A, arrows) and lower solidity (Figure 2B) in the stiff gel indicate a different phenotype. Though the phenotype changed, there is no observable difference that the stiffened matrix caused cell cycle arrest (Figure S2A, Supporting Information). We also confirmed that matrix stiffening did not affect macrophage survival, apoptosis, or proliferation (Figure S2B,C, Supporting Information).

To further determine the phenotypic change of BMDMs in a stiffening environment, we characterized the expression of CD206, a marker associated with the pro‐regenerative phenotype, and pro‐tumoral immunosuppressive tumor‐associated macrophages (TAM).^[^ ^44^ ^]^ Further, we also evaluated the expression of major histocompatibility class II molecules (MHC‐II), which are expressed at higher levels in macrophages exposed to inflammatory stimuli compared to non‐activated cells,^[^ ^45^, ^46^ ^]^ and in immunostimulatory TAM.^[^ ^47^ ^]^ Immunofluorescent staining found more CD206^+^ BMDMs in the stiff gel compared to the soft matrix (Figure 2C). Flow cytometry analysis confirmed this finding, showing that BMDM with a pro‐regenerative phenotype (CD206^+^MHC‐II^low^), and the ratio of pro‐regenerative over pro‐inflammatory BMDMs (CD206^+^MHC‐II^low^/CD206^−^MHC‐II^high^) were significantly increased upon matrix stiffening (Figure 2D; S2D, Supporting Information). We also quantified the absolute cell number in each group by flow cytometry showing that the stiffer matrix had a significantly higher CD206^+^MHC‐II^low^ cell number than the soft matrix (Figure S2E, Supporting Information). Further, we confirmed that the higher collagen concentration in the stiff hydrogels did not affect macrophage phenotype (Figure S1E, Supporting Information), suggesting that BMDM polarization was caused predominantly by matrix stiffening rather than changes in matrix composition.

Next, we treated BMDMs with interleukin 4 (IL‐4, 20 ng mL^−1^), a cytokine that stimulates pro‐regenerative M2‐like polarization.^[^ ^48^ ^]^ We observed that starting from soft gel, stiffening (stiff w/o IL‐4 group) or adding IL‐4 (soft w/ IL‐4 group) yielded similar CD206^+^MHC‐II^low^ population and CD206^+^MHC‐II^low^/CD206^−^MHC‐II^high^ ratio (Figure 2D; Figure S2D, Supporting Information), indicating a similarity between cytokine and mechanically induced polarization. IL‐4 did not cause cell cycle arrest while inducing polarization (Figure S2F, Supporting Information). Further, in the presence of optimal IL‐4 concentration (>2 ng ml^−1^), the change in matrix stiffness did not have an additional effect on BMDM's phenotype, suggesting that in our experimental conditions, tissue stiffness phenocopy the effects of optimal IL‐4 concentrations on the macrophage popularization program (Figure S2G, Supporting Information).

Finally, we performed a real‐time RT‐qPCR analysis to characterize the gene expression related to different macrophage phenotypes (Figure 2E). We observed that the expression of genes typically associated with pro‐regenerative macrophages,^[^ ^44^ ^]^ including *Retnla*, *Chil3*, and *Lgals3*, were stimulated when stiffening was induced. On the contrary, pro‐inflammatory genes such as *Irf7*, *Tlr7*, and *Ifit3* were downregulated in the stiff group compared to the soft group. A comparison between the soft+IL‐4 and stiff groups showed that IL‐4‐induced and stiffening‐induced polarization resulted in similar effects regarding the expression of phenotype‐related genes. These results support our conclusion that matrix stiffening phenocopies the effects of IL‐4 stimulation, polarizing BMDMs toward a pro‐regenerative phenotype.

### 2.3 Transcriptomics Analysis Reveals Programming of the BMDMs in Dynamically Stiffened Hydrogel

**Figure 3:**
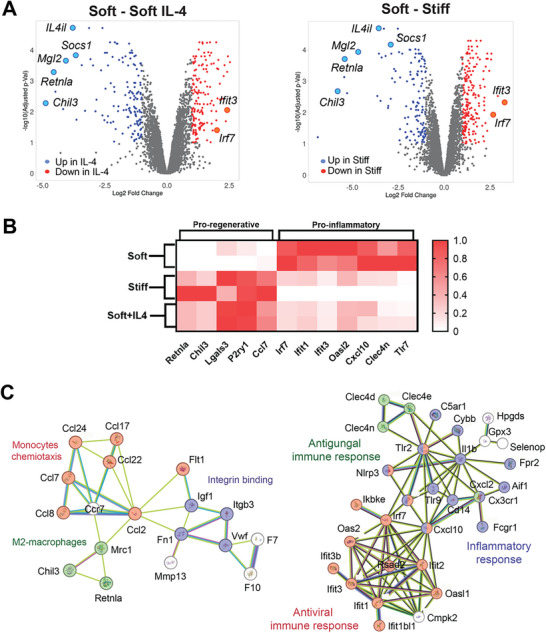
Transcriptomics analysis of the BMDMs in dynamically stiffened hydrogel. A) Volcano plots of the differentially expressed genes (DE) in the stiff and soft+IL‐4 groups compared with the soft group. B) Heatmap of DE genes associated with pro‐regenerative and inflammatory response. C) Network analysis of DE upregulated (left) and downregulated (right) in soft+IL‐4 and stiff hydrogel BMDM.

Next, we sought to characterize the effects of IL‐4 and matrix stiffness on the BMDM global transcriptomics program. For this purpose, RNA sequencing was performed on BMDM cultured for 48 h in soft matrix with or without an optimal concentration of IL‐4, or cultured in stiff matrix alone. Gene expression analysis indicated that 142 differentially expressed (DE) genes were significantly downregulated and 149 upregulated in BMDM cultured in the presence of IL‐4 response (p‐adjusted value = 0.05; Log_2_[fold change] = 2; Figure S3A, Supporting Information). Similarly, in response to the stiff matrix, 142 and 149 DE genes were found downregulated or upregulated, respectively (Figure S3A, Supporting Information). In the soft matrix, exposure to IL‐4 stimulates the expression of genes typically associated with the M2‐like/pro‐regenerative phenotype, while inducing a parallel downregulation of interferon‐stimulated genes (**Figure** **3**A, left). Interestingly, culturing BMDM in stiff matrix alone induces similar changes, overlapping the effects of IL‐4 on BMDM cultured in soft hydrogel (Figure 3A, right). Specifically, genes associated with M2‐macrophage polarization (e.g., *Retnla*, *Chil3*, and *Mrc1*), monocyte chemotaxis (e.g., *Ccl17* and *Ccl22*), and integrin binding (e.g., *Itgb3* and *Fn1*) were upregulated by culturing BMDM in soft matrix with IL‐4 or stiff matrix (Figure 3B,C, left). On the contrary, genes associated with the anti‐viral, anti‐fungal, and inflammatory response were found to be downregulated in response to IL‐4 or stiff matrix (Figure 3B,C, left). In total, 114 and 86 DE genes were found downregulated and upregulated, respectively, in response to IL‐4 or stiff matrix (Figure S3B, Supporting Information), suggesting a similar activation program. Pathway analysis further confirmed this finding, showing that culture in the stiff matrix or IL‐4 induced a positive enrichment of genes associated with pro‐regenerative pathways (e.g., oxidative phosphorylation, glycolysis/gluconeogenesis). On the contrary, pathways associated with antiviral response and inflammation (e.g., Hepatitis C, Toll‐like receptor, RIG‐I‐like receptor) were downregulated in BMDM stiff (Figure S3C, Supporting Information). Lastly, although a few genes were found differentially regulated by IL‐4 or stiff matrix, no DE genes were identified by comparing BMDM generated in IL‐4 with those cultured in stiff hydrogel (Figure S3A,B, Supporting Information). This finding corroborates and extends the results from phenotypic and flow cytometry analyses, demonstrating that adaptation to stiff microenvironment phenocopies the effects of IL‐4, driving the pro‐regenerative activation program in BMDM.

### 2.4 CaMKK2 Mediates Stiffness‐Induced BMDM Polarization

Next, we sought to elucidate the mechanism of stiffness‐induced BMDM polarization. Our previous study indicated that loss of CaMKK2 impairs the activation program of macrophages by interfering with cross‐talk between integrin and toll‐like receptor signaling.^[^ ^30^ ^]^ More recently, we also demonstrated that CaMKK2 expressed in myeloid cells has an important role in the mechanism regulating the immunosuppressive TME, and blocking this kinase unleashes the anti‐tumor immune response.^[^ ^33^, ^49^ ^]^ Further, other studies showed that the CaMKK2 pathway is mechanosensitive and is strongly correlated with cell‐matrix interactions.^[^ ^50^ ^]^ Therefore, we interrogate whether the mechanical modulation of BMDMs in our 3D stiffening matrix depends on CaMKK2.

**Figure 4:**
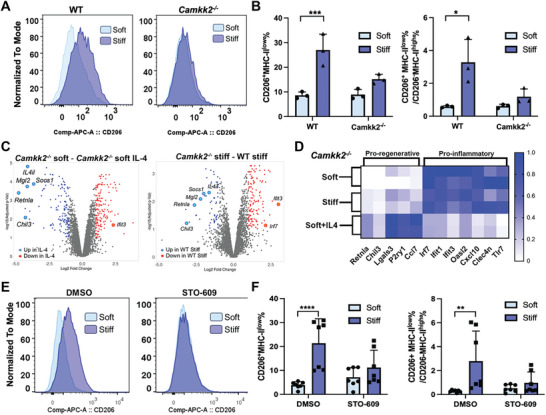
Role of Camkk2 in stiffness‐induced BMDM polarization. A,B) Representative flow cytometry histograms and quantification of analysis of CD206 expression on CD11b^+^F4/80^+^ gated *Camkk2^−/−^* and WT BMDMs encapsulated in the hydrogels after 2 days. N = 3 (*t*‐test, *p<0.05, ***p<0.001). C,D) Bulk RNA sequencing analysis of *Camkk2^−/−^* BMDMs in soft, stiff, and soft+IL‐4 hydrogels. E,F) Representative flow cytometry histograms and quantification of CD206 expression on CD11b^+^F4/80^+^ gated BMDMs treated with DMSO (negative control) and STO‐609 (CaMKK2i) encapsulated in the hydrogels after 2 days. N = 3 (*t*‐test, **p<0.01, ****p<0.0001).

We first examined the phenotypic changes of BMDMs from *Camkk2*‐deficient mice in different stiffness conditions. *Camkk2^−/−^* BMDMs showed negligible polarization toward CD206^+^MHCII^low^ cells when stiffness increased (**Figure** **4**A). Quantification validated that matrix stiffening did not significantly increase the CD206^+^MHC‐II^low^ population or CD206^+^MHC‐II^low^/CD206^−^MHC‐II^high^ ratio in *Camkk2^−/−^* BMDMs, as opposed to the results from wild type (WT) BMDMs (Figure 4B).

Transcriptomic analysis of *Camkk2* ^−/−^ BMDMs indicated that the genetic ablation of Camkk2 did not interfere with the activation program of BMDMs cultured in the soft hydrogel, which showed a gene signature superimposable with WT BMDMs cultured in the same conditions (Figure 4C, D; Figure S4A,B, Supporting Information). On the contrary, we found remarkable differences in the transcriptomic profiles of WT and *Camkk2* ^−/−^ BMDMs cultured in stiff hydrogels (Figure S4A,B, Supporting Information). Enrichment and network analysis demonstrated that genes downregulated in *Camkk2* ^−/−^ compared to WT BMDMs cultured in the stiff matrix were associated with “M2‐macrophages” and “monocytes migration”, while genes associated with “antiviral” and “inflammatory” responses were upregulated in *Camkk2* ^−/−^ BMDMs (Figure S4C, Supporting Information). Consistently, a few DE genes were identified by comparing the transcriptomic profiles of *Camkk2* ^−/−^ BMDMs cultured in soft or stiff hydrogels (Figure S4D, Supporting Information). The heatmap demonstrated that regardless of pro‐regenerative or pro‐inflammatory, most phenotype‐related genes were expressed at comparable levels in the different stiffness conditions (Figure 4D). However, the soft+IL‐4 group showed a substantially increased pro‐regenerative gene expression and a downregulated pro‐inflammatory gene expression. The same trend was observed in the gene expression in *Camkk2^−/−^* BMDMs measured by RT‐qPCR (Figure S4E, Supporting Information). These data proved that Camkk2 has a pivotal role in the mechanically‐induced macrophage fate signaling, while deletion of this kinase does not affect the ability of IL‐4 to drive BMDM pro‐regenerative polarization. An inhibition study using STO‐609,^[^ ^51^ ^]^ a selective and cell‐permeable inhibitor of CaMKK2, found that STO‐609‐treated BMDMs had no phenotypic change when matrix stiffness increased (Figure 4E). Quantification validated that matrix stiffening did not significantly increase the CD206^+^MHC‐II^low^ population or CD206^+^MHC‐II^low^/CD206^−^MHC‐II^high^ ratio in BMDMs treated with STO‐609, as opposed to the results of the DMSO control group (Figure 4F). This data further supports that CaMKK2 mediates mechanically‐induced macrophage fate and provides a proof‐of‐concept for pharmacologically targeted inhibition of CaMKK2 to diminish stiffness's impact on modulating macrophage fate.

To investigate the expression and activation state of CaMKK2 under different matrix stiffness conditions, we utilized a CaMKK2‐EGFP reporter mice model and studied BMDMs from CaMKK2‐EGFP mice in soft and stiff hydrogels. Our results showed no significant differences in EGFP expression between the cells cultured in soft and stiff hydrogels (Figure S4F, Supporting Information), suggesting that the CaMKK2 promoter activity is not notably influenced by changes in hydrogel stiffness.

### 2.5 Stiffening‐Induced Pro‐Tumoral Macrophage Polarization

**Figure 5:**
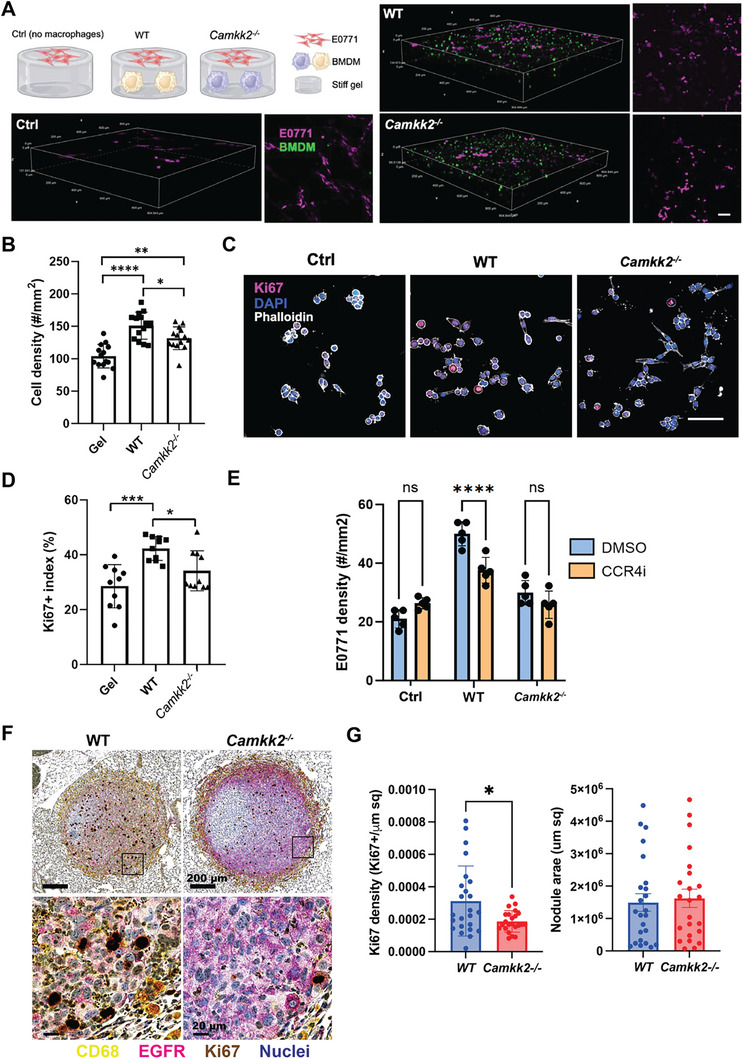
Camkk2 controls pro‐tumoral functions of macrophage induced by stiff matrices. In vitro studies: A) Tumor cell (EO771) and macrophage (BMDM) co‐culture experiments. EO771 cells (IRFP‐labeled) were seeded on top of the stiff Col‐HA hydrogel encapsulated with or without BMDMs (GFP‐labeled, WT, or *Camkk2* ^−/−^). Fluorescent images were taken 2 days after seeding the tumor cells. Scale bar = 50 µm. B) Quantification of EO771 cell density. N = 3 with 15 images in each group (One‐way ANOVA, *p<0.05, **p<0.01, ****p<0.0001). C) Representative confocal images of EO771 cells stained with Ki67 (magenta) and DAPI (blue). Scale bar = 50 µm. D) Quantification of Ki67 positive cell percentage. N = 3 with 10 images in each group (One‐way ANOVA, *p<0.05, ***p<0.001). E) Quantification of EO771 cell density on top of the hydrogels encapsulated with or without BMDMs (WT or *Camkk2* ^−/−^) and treated with DMSO or CCR4 inhibitor. N = 3 with 5 images in each group (One‐way ANOVA, ****p<0.0001). F,G) In vivo studies: EO771‐Luc cells (200000 cells/mouse) were inoculated in the tail vein of WT and *Camkk2^−/‐^* mice and animals were euthanized 30 days after tumor cell injection (N = 5 mice/genotype). Lungs were removed, and their histological sections were stained to identify myeloid cells, tumor cells, and proliferating cells, using antibodies against CD68, EGFR, and Ki67 (Yellow, purple, and brown nuclear staining, respectively). Nuclei were identified by hematoxylin staining (blue). F) Representative staining of tumor nodules identified in WT and *Camkk2^−/‐^* mice. The top and bottom images refer to high and low magnification, respectively. Squares in the top panels indicate magnified areas shown in the bottom panels. G) Ki67⁺ density and lung tumor nodules surface. Each data point refers to Ki67 density measured in a single tumor nodule. At least 2 nodules for each tumor were analyzed. *T*‐tests, *p< 0.01.

We have validated that an increase in matrix stiffness stimulates macrophage polarization into CD206^+^MHC‐II^low^ cells. Such a phenotype is generally recognized as immuno‐suppressive or pro‐tumoral in the TME. While the accumulation of MHC‐II^high^ TAM has been associated with a better clinical outcome,^[^ ^52^ ^]^ ECM stiffness is emerging as an important factor that regulates critical functions of infiltrating immune cells and, in turn, tumor growth.^[^ ^53^ ^]^ Using global or myeloid‐specific conditional mice in conjunction with breast cancer, lymphoma, and glioblastoma models, we previously demonstrated that deletion of *Camkk2* in the host resulted in increased accumulation of inflammatory TAM, enhanced anti‐tumor immune response, and inhibition of the tumor growth.^[^ ^33^, ^34^, ^49^ ^]^ We established that the deletion of *Camkk2* in macrophages enhances the CD8^+^ T cell‐mediated anti‐tumor response by upregulating genes involved in inflammatory responses (e.g., *Tnfa*) and T cell recruitment (e.g., *Cxcl10*), and downregulating MHC II expression, with minor effects on genes typically associated with M2‐like polarization, such as *Retnla* and *Mrc1*.^[^ ^32^ ^]^ In this work, we are interested in investigating the effect of macrophages polarized by environmental factors on sustaining tumor growth and the role of *Camkk2* in such a process. With this in mind, we leveraged our dynamic hydrogel model to investigate whether stiffening‐induced macrophage polarization affects tumor cell growth. We seeded EO771, a mouse mammary fat pad–derived adenocarcinoma cell line, on top of the stiff Col‐HA hydrogels (**Figure** **5**A). We found that when BMDMs were encapsulated in the hydrogel, the tumor cell density significantly increased compared with that in the hydrogel‐only group (Figure 5A,B), suggesting that macrophages in the matrix promoted tumor cell growth. Furthermore, we found increased tumor cell density in hydrogels encapsulating WT BMDMs compared to *Camkk2^−/−^* BMDMs (Figure 5A–C). We also observed that tumor cells were more proliferative in the presence of WT BMDM, with a ≈10% higher Ki67 index than *Camkk2^−/−^* BMDMs (Figure 5C,D).

In the TME, macrophages secrete various chemokines and cytokines to regulate tumor progression. Among them, CCL17 and CCL22 have been reported to be secreted at a high‐level in TAM.^[^ ^54^ ^]^ High expression of these chemokines in the TME has direct and indirect pro‐tumoral effects, stimulating the proliferation of tumor cells while favoring the accumulation of immunosuppressive cells in TME.^[^ ^55^, ^56^ ^]^ On the other hand, the C‐C chemokine receptor 4 (CCR4), which binds CCL17 and CCL22, and is highly expressed in several types of tumors, is emerging as an important druggable target to control tumor growth and promote the anti‐tumor immune response.^[^ ^57^ ^]^ Our RNA sequencing data showed that WT BMDMs had an upregulated CCL17 and CCL22 expression than *Camkk2^−/−^* BMDMs in the stiff hydrogel (Figure S4D, Supporting Information). We thus reasoned that these chemokines could directly stimulate the growth of EO771 tumor cells and tested this hypothesis by using a small molecule CCR4 antagonist. The results of these experiments confirmed our hypothesis, indicating that blockade of CCR4 significantly reduced tumor cell growth when EO771 cells were co‐cultured with WT BMDMs. On the other hand, such an effect was negligible when tumor cells were co‐cultured with *Camkk2^−/−^* BMDMs (Figure 5E). In addition to these chemokines, we also demonstrated the expression of *Cxcl10*, a critical inflammatory gene, was downregulated in stiffer environments (Figure 3); and the lack of *Camkk2* counteracts the inhibitory effect of stiffness on *Cxcl10*, thereby impairing the ability of BMDM in the stiff matrix to sustain the tumor growth. In aggregate, data from the hydrogel model highlights the role of Camkk2 in the mechanism coupling mechanosensory response and pro‐tumoral activity of macrophages in the TME, paving the way for a translation use of 3D hydrogel co‐culture for drug screening and development.

We originally identified Camkk2 expressed in tumor stromal cells as a critical component of molecular signaling controlling the immunosuppressive microenvironment in primary tumors.^[^ ^33^, ^34^, ^49^ ^]^ The cancer‐cell extrinsic function of CaMKK2 has been validated by others, which demonstrated that *Camkk2* expressed in the host cells supports the growth of pancreatic adenocarcinoma cells,^[^ ^58^ ^]^ and impairs metastatic colonization of prostate cancer cells.^[^ ^59^ ^]^ Mechanistically, we demonstrated that deletion of *Camkk2* in the host, particularly in myeloid cells, unleashes the anti‐tumor immune response, inhibiting the growth of EO771 cells in the mammary gland fat pad.^[^ ^49^ ^]^ Noteworthy, data from the hydrogel model suggests that, alongside this immunomodulatory effect, *Camkk2* expressed in the host cells can also directly regulate the proliferation of tumor cells. To test this hypothesis, we used *Camkk2^−/−^* mice in conjunction with a metastatic model of mammary tumor cells. EO771 tumor cells expressing a bioluminescent reporter gene (EO771‐Luc) were thus inoculated in the tail vein of WT and *Camkk2^−/−^* mice, and the whole‐body metastatic burden was monitored by bioluminescence. The deletion of *Camkk2* in the host cells restrained lung metastasis development (Figure S5A, Supporting Information), enhancing the survival of *Camkk2^−/−^* compared to WT (Figure S5A,B, Supporting Information). To determine whether this effect correlated with decreased tumor cell proliferation, we replicated this experiment using an independent set of mice. These animals were euthanized 30 days after tail vein inoculation of EO771‐Luc, the lungs infiltrated by tumors showing a comparable size were collected, and multiplex stained to identify epithelial tumor cells (epidermal growth factor receptor, EGFR^+^), myeloid cells (CD68^+^), and proliferating cells (Ki67^+^). The density of Ki67^+^ tumor cells in nodules was then assessed using QuPath software.^[^ ^60^ ^]^ Interestingly, a higher density of Ki67^+^ cells was detected in the lung tumor nodules growing in WT mice compared to *Camkk2^−/−^* mice (Figure S5C, Supporting Information; Figure 5F,G). This result confirms that the deletion of *Camkk2* in host cells is associated with an increased proliferation rate of tumor cells, validating our hydrogel model of TME for translation applications.

### 2.6 Mechano‐CaMKK2‐Dependent Modulation of Immune Cell Response in Wound Healing

**Figure 6:**
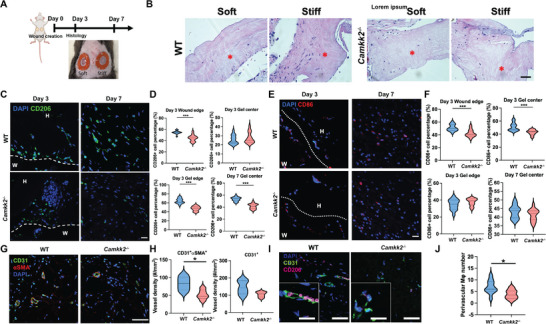
Immune and inflammatory response of the implantation of Col‐HA hydrogels to mouse excisional wounds. A) Timeline of the animal experiments. B) H&E images of the wound on day 3. Scale bar = 50 µm. Red stars indicate hydrogels. C) Representative fluorescent images of CD206 staining on day 3 and day 7. H = hydrogel, W = wound. Scar bar = 25 µm. D) Quantification of CD206^+^ cell density at the wound edge, gel edge, and gel center on day 3 and day 7. N = 3 with 15 measurements per group (*t*‐test, ***p<0.001). E) Representative fluorescent images of CD86 staining on day 3 and day 7. H = hydrogel, W = wound. Scar bar = 25 µm. F) Quantification of CD206^+^ cell density at the wound edge, gel edge and gel center on day 3 and day 7. N = 3 with 15 measurements per group (*t*‐test, ***p<0.001). G) Representative fluorescent images of CD31 and αSMA staining on day 7 to characterize the vascularization. Scale bar = 50 µm. H) Quantification of CD31^+^αSMA^+^ and CD31^+^ blood vessel density. N = 3 with 5 measurements per group (*t*‐test, *p<0.05). I) Representative fluorescent images of CD31 and CD206 staining on day 7 to characterize perivascular macrophage distribution. Scale bar = 50 µm (Insert: scale bar = 20 µm). J) Quantification of perivascular CD206^+^ cell density. N = 3 with 10 measurements per group (*t*‐test, *p<0.05).

During wound healing, macrophages can infiltrate the wound to combat an infection and facilitate the healing process. The function and phenotype of macrophages shift dynamically throughout different stages of wound healing, including inflammatory, proliferative, and resolution phases.^[^ ^61^ ^]^ The phenotypic changes are essential for the proper orchestration of the complex healing process.^[^ ^4^ ^]^ Thus, we sought to investigate whether local stiffness would affect macrophage fate and further direct immune activities such as immune cell recruitment and inflammatory response. We chose a mouse excisional wound healing model as the initial inflammatory stage after injury, which involves various immune cells, especially macrophages, to restore tissue homeostasis in the healing process.^[^ ^62^ ^]^ We created wounds on the dorsal skin of WT and *Camkk2^−/−^* mice, treated them with soft or stiff hydrogels (**Figure** **6**A) and evaluated cell‐ECM interaction and inflammatory response. In H&E staining, almost entirely intact hydrogels were visible on day 3, with little cell infiltration into the hydrogel (Figure 6B; Figure S6A, Supporting Information). On day 7, extensive cell‐matrix interactions were seen with fragmented hydrogel, and the boundaries between gel and local tissue faded (Figure S6B, Supporting Information). Comparing the wounds in WT and *Camkk2^−/−^* animals, we found that cell infiltration and cell‐ECM integration were faster in WT, suggesting that the wound healing in WT animals progressed from inflammatory to regenerative phase faster compared with *Camkk2^−/−^* animals.

We further characterized the phenotype of macrophages in the wound. We focused on stiff hydrogels because the in vitro data indicated that distinct WT and *Camkk2^−/−^* macrophage phenotypes were only observed in stiff hydrogels. Since the stiffening matrix only modulates the fate of WT macrophages, but not *Camkk2^−/−^* macrophages, we expected that the native macrophages in WT and *Camkk2*‐deficient animals would react differently to the stiff hydrogel. On day 3, the tissue‐hydrogel boundary was clear. Hence, we analyzed the macrophages at the wound edge, hydrogel edge, or hydrogel center. However, as on day 7 the hydrogel had been integrated with native tissues, we could analyze only the macrophages in the center of the tissue‐hydrogel complex. We found that the percentage of CD206^+^ macrophage was significantly higher in WT mice than in *Camkk2^−/−^* mice on both day 3 (wound edge and hydrogel edge) and day 7 (Figure 6C,D). CD86 staining showed that the rate of pro‐inflammatory macrophage at the wound‐hydrogel boundary was higher in WT mice than in *Camkk2^−/−^* mice, but such a trend was only observed on day 3 (Figure 6E,F).

Finally, we evaluated if different macrophage subphenotypes in WT and *Camkk2^−/−^* mice affected vascularization. Assessing CD31 and αSMA, we found more CD31^+^ vessels and CD31^+^αSMA^+^ vessels in WT mice than in *Camkk2*‐deficient mice (Figure 6G,H). To verify if pro‐regenerative macrophages participated in vascularization, we stained CD31 and CD206 to identify perivascular pro‐regenerative macrophages. We found that the WT animals had a significantly higher CD206^+^ cell density near CD31^+^ lumens than *Camkk2^−/−^* animals (Figure 6I,J), suggesting that the stiff hydrogels regulated macrophage polarization toward a pro‐regenerative phenotype, further facilitating vascularization. However, vascularization was slower in the *Camkk2^−/−^* mice, indicating that the mechanical modulation depends on CaMKK2.

### 2.7 CaMMK2 Regulates Stiffness‐Induced BMDM Polarization Through AKT/AMPK‐STAT6 Signaling

We next sought to elucidate the mechanism related to CaMKK2 that dictates macrophage fate in a stiffened microenvironment. Since CaMKK2 is a critical mediator in the calcium ion signaling, we assessed the ionic concentrations within the hydrogels under different stiffness conditions. We tested the hydrogels without cells to examine whether environmental ionic concentrations are affected by matrix stiffness, which could further influence cellular CaMKK2 activity. We found that the Ca^2+^ concentrations in the media (supernantant) were similar in soft and stiff gels, and are much higher than the results measured in the hydrogels (Figure S7A, Supporting Information). This is because the media we used contained bovine serum. Thus, we further washed the hydrogel to exclude Ca^2+^ in the media and tested local ionic concentration, which has a direct impact on cellular activity. We also observed a similar Ca^2+^ concentration in soft and stiff hydrogels. These data confirm that different matrix stiffness conditions will not cause variations in ionic concentrations in the microenvironment where macrophages reside.

**Figure 7:**
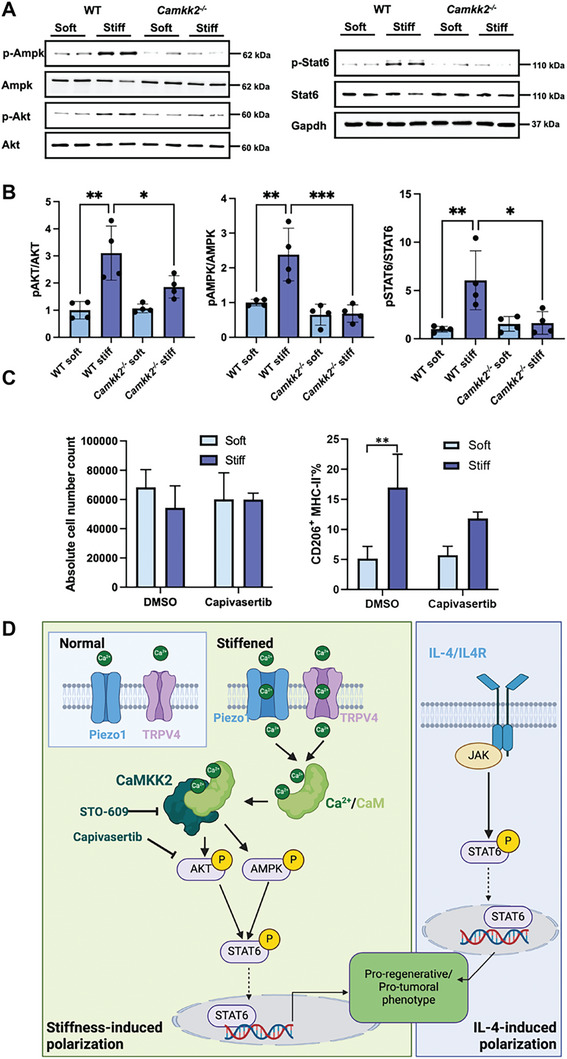
Mechanistic study of stiffness‐induced CaMKK2‐dependent BMDM polarization. A) Western blot to analyze (phosphorylated) Akt, Ampk, and Stat6 expression in different conditions. Gapdh was used as a protein loading control. D) Quantification of Western blot. The ratio of the expression of phosphorylated protein to total protein was quantified and normalized to the WT soft group. N = 4. (One‐way ANOVA, *p < 0.05, **p < 0.01, ***p<0.001). C) Flow cytometry analysis of BMDMs treated with DMSO (control) and Capivasertib (Akt inhibitor). Absolute total macrophage number and CD206^+^MHC‐II^low^ percentage were quantified. N = 3. (*t*‐test, **p < 0.01). D) Proposed mechanisms of stiffness‐induced macrophage fate change. Matrix stiffening opens mechanosensitive ion channels and causes the increased influx of Ca^2+^ ions, which activates CaMKK2 and its downstream activators (i.e., AKT, AMPK). These signaling proteins eventually facilitate STAT6 phosphorylation and translocation to the nucleus and direct macrophage polarization toward a pro‐regenerative/pro‐tumoral phenotype. In parallel, IL‐4 induced a similar polarization through JAK/STAT6 signaling independent of CaMKK2.

Then, we investigated the downstream signaling pathways. CaMKK2 links calcium signaling with the downstream activation of AMP‐activated protein kinase (AMPK),^[^ ^63^, ^64^ ^]^ a master metabolic regulator, regulating macrophage polarization,^[^ ^65^, ^66^, ^67^, ^68^ ^]^ and mechanosensory signaling.^[^ ^50^ ^]^ Further, AKT (also known as protein kinase B) has been identified as a downstream target of CaMKK2 in tumor cells.^[^ ^69^ ^]^ We first tested the effects of stiffness and *Camkk2* deletion on Ampk phosphorylation. Western blot analyses showed that a stiffer matrix was sufficient to stimulate Ampk phosphorylation in WT BMDMs (**Figure** **7**A,B). On the contrary, *Camkk2*‐deficient BMDMs showed diminished Ampk phosphorylation, regardless of the matrix stiffness. We previously reported that CaMKK2 is required for the activation of the AMPK pathway to regulate the fate of myeloid‐derived suppressor cells.^[^ ^33^ ^]^ Here we extended this observation, demonstrating that in BMDMs, CaMKK2 links the macrophage mechanosensory response to AMPK phosphorylation. We next investigate the effects of CaMKK2 deletion on AKT signaling, which has been reported to modulate macrophage polarization.^[^ ^70^, ^71^ ^]^ The activation of AKT by CaMKK2 has been documented in cancer cells.^[^ ^69^, ^72^ ^]^ In addition, we originally found a decreased accumulation of phosphorylated Akt in lipopolysaccharides (LPS)‐stimulated *Camkk2^−/−^* BMDM compared to WT.^[^ ^30^ ^]^ Western blot analyses further showed that stiffer matrix stimulated Akt phosphorylation in WT BMDMs (Figure 7A), with 3 times increase in p‐Akt/Akt ratio (Figure 7B). However, no significant difference in the p‐Akt/Akt ratio between the two stiffness conditions was observed in *Camkk2^−/−^* BMDMs, suggesting that Akt activation in stiff matrices is dependent on Camkk2.

Since AKT regulates critical function in tumor cells, this protein is emerging as a clinically relevant target in cancer therapy.^[^ ^73^ ^]^ Alongside the direct effect on tumor cells, blocking of AKT stimulates T cell functions, promoting the expansion of tumor‐specific lymphocytes.^[^ ^74^ ^]^ Our data suggests that AKT blockers would also influence the TME by preventing the generation of M2‐like/pro‐tumoral macrophages, thereby favoring the anti‐tumor immune response while inhibiting the pro‐tumoral effects of these cells. To explore this clinically relevant hypothesis, we used Capivasertib, an FDA‐approved AKT inhibitor.^[^ ^75^ ^]^ We found that Capivasertib treatment did not affect the total macrophage number (Figure 7C, left). However, treatment with this drug was associated with a decreased percentage of the CD206^+^MHC‐II^low^ subset (Figure 7C, right). This data indicates confirmed the role of CaMKK2‐AKT signaling in the mechanism coupling the mechanosensory response to the induction of M2‐like polarized BMDMs, revealing a novel potential therapeutic use of Akt blockers to modulate macrophage polarization in stiff tissues, such as those found in tumors and fibrosis.

The signal transducer and activator of transcription protein‐6 (STAT6) is a well‐known downstream effector of IL‐4 signaling and a master regulator of the M2‐like polarization process.^[^ ^76^ ^]^ Once phosphorylated in response to IL‐4 stimulation, STAT6 translocates to the nucleus to activate the transcriptional program leading to an M2‐like phenotype.^[^ ^77^ ^]^ AKT and AMPK signaling also contribute to this process by phosphorylating STAT6.^[^ ^68^, ^71^ ^]^ Additionally, recent evidence indicates that matrix rigidity stimulates the nuclear import of STAT6, facilitating the polarization of macrophages toward the M2‐like phenotype.^[^ ^78^ ^]^ We thus examined whether exposure to a stiff matrix was sufficient to trigger Stat6 phosphorylation and the role of Camkk2 in this pathway. The results of these experiments indicated that stiffness alone was sufficient to stimulate the phosphorylation of Stat6 in WT BMDMs. In contrast, the loss of Camkk2 completely abrogated this phenomenon, indicating that in macrophages Camkk2 is required to link mechanosensory signals to Stat6‐mediated signaling (Figure 7C,D). Both IL‐4 and matrix stiffening‐induced Stat6 phosphorylation explain the overlapping phenotype of BMDM generated in soft gel in the presence of IL‐4 and those cultures in stiff matrix alone. Importantly, our data identifies Camkk2 as a crucial node that selectively regulates stiffness‐induced signaling. At the same time, deletion of *Camkk2* does not impair the ability of BMDM to polarize toward the M2‐like pathway, in response to IL‐4 stimulation. In myeloid cells, ion channels like Piezo1 and TRPV4, alongside other membrane receptors such as integrins, can capture the mechanical forces in a stiffened matrix, triggering calcium transients^[^ ^79^ ^]^ to activate CaMKK2.^[^ ^27^, ^28^, ^29^ ^]^ In our model, CaMKK2 activation phosphorylates AMPK and AKT and, in turn, stimulates STAT6 phosphorylation and translocation to the nucleus to trigger the transcriptional program driving macrophage polarization toward a pro‐regenerative/pro‐tumoral phenotype (Figure 7D, left part). In parallel, IL‐4 induces a similar polarization through Janus kinase (JAK)/STAT6 signaling but independent of CaMKK2 (Figure 7D, right part), as evidenced by the Western blot results that IL‐4 enhanced Stat6 phosphorylation regardless of Camkk2 (Figure S7B, Supporting Information). Although further studies are required to fully define the role of CaMKK2 downstream targets, such as AMPK and AKT, in this mechanism, our results open interesting translational perspectives for shaping the macrophage landscape in normal and pathological tissues by targeting CaMKK2.

## 3 Discussion

Macrophages have been shown to sense the stiffness of the environment and initiate polarization. Still, the literature has not agreed on whether a stiffer environment favors an M1 or M2 phenotype. Some studies suggested that a stiff environment promotes M1 polarization, mimicking the inflammatory conditions associated with diseases like fibrosis and atherosclerosis.^[^ ^15^, ^17^, ^21^, ^23^ ^]^ In contrast, other studies proposed that a stiff matrix favors M2 polarization, resembling the tissue repair and remodeling processes.^[^ ^16^, ^18^ ^]^ Various factors can contribute to these discrepancies: (1) these studies utilized 2D substrates with varying stiffness, which does not fully recapitulate the macrophage niche in the physiological environment; (2) the “stiff” matrix is defined differently in the literature, ranging from 1 to 1000 kPa; (3) variability of macrophage cells used in the different experiments, including transformed or tumor cell lines (e.g., RAW 264.7, U937), BMDM, monocyte‐derived macrophages; (4) Parameters used to define M1/M2 phenotype (e.g., phenotype, transcriptomics, functions), which cannot precisely characterize macrophage phenotype in varying environments.^[^ ^6^ ^]^ Here, we used primary BMDMs, which were generated using a low‐attachment plate to prevent their terminal differentiation, thus preserving their polarization potential.^[^ ^80^ ^]^ In addition, we adopted a collagen‐based hydrogel capable of dynamic stiffening to study macrophage fate in 3D. The Young's modulus of the hydrogel is less than 0.5 kPa, mimicking the stiffness of soft tissues such as adipose, lung, brain, and bone marrow.^[^ ^81^ ^]^ After stiffening, Young's modulus increased to ≈2 kPa, modeling the diseased (fibrosis, TME) state of these tissues.^[^ ^82^ ^]^ We find that such an increase in matrix stiffness drives macrophage polarization toward a CD206^+^MHC‐II^low^ phenotype, resembling pro‐regenerative and immunosuppressive TAM. The transcriptomic analyses reveal that such phenotype is associated with regeneration (oxidative phosphorylation and glycolysis) and downregulated antiviral response and inflammation, suggesting that an increase in matrix stiffness can activate macrophages, suppress inflammatory response, and promote tumor progression or regeneration.

The diverse functions of macrophages are shaped by their phenotype. Therefore, modulating macrophage fate in a diseased environment has become an evolving direction that profoundly impacts the treatment of various diseases. For example, in wound healing, it has been well elucidated that tissue‐resident dermal macrophages respond to biochemical signals and shift their phenotypes to orchestrate immune activities and wound repair.^[^ ^4^, ^61^ ^]^ Yet the role of mechanical cues, such as stiffness, is often overlooked in this process. Here, we show that the phenotype and function of native macrophages are affected by ECM stiffness. The stiffened hydrogel promotes local immune cell engraftment and integration with the matrix in the early stage, which can be attributed to its collagen‐based nature and its similar stiffness to reticular dermis tissue (i.e., 1–2 kPa).^[^ ^83^ ^]^ The stiffened hydrogel further polarizes the recruited macrophages toward a pro‐regenerative phenotype to aid vascularization and wound repair in the long term, which is consistent with our findings in vitro that BMDMs encapsulated in stiffened hydrogel polarize toward CD206^+^MHC‐II^low^ subpopulation. It is also noteworthy that the concepts of “soft” and “stiff” here are relative and context‐dependent. Future work needs to be done to delineate macrophage phenotype across a wider range of stiffness levels, thereby expanding our knowledge of macrophage behavior throughout the body. Nevertheless, our findings shed light on engineering mechanical cues in a diseased microenvironment to manipulate macrophage fate to facilitate therapeutic regeneration.

Although many studies have been conducted to elucidate the intricate interplay between macrophage phenotype and matrix stiffness, the underlying molecular mechanism has yet to be discovered. Here, we show that stiffening‐induced macrophage polarization produces a phenotype similar to IL‐4‐induced polarization. IL‐4 is an essential M2 polarizing cytokine that acts through IL‐4Rα/JAK/STAT6 signaling.^[^ ^76^ ^]^ Interestingly, we show that stiffness‐induced macrophage polarization is also associated with STAT6 phosphorylation. However, our data indicates that stiffness and IL‐4 act through different upstream signaling. In stiffness‐induced macrophage polarization, we identify CaMKK2 as a key component linking stiffness‐induced signaling to STAT6 activation and M2‐like polarization. On the other hand, our data also indicates that CaMKK2 is dispensable for IL‐4‐induced M2‐like polarization. CaMKK2 is activated by increased Ca^2+^ influx, while a variety of receptors may contribute to triggering Ca^2+^ transient in the stiff matrix. Several ion channels on macrophages, such as Piezo1^[^ ^22^, ^84^ ^]^ and TRPV4,^[^ ^85^ ^]^ are mechanosensitive and can open in response to mechanical stimuli in a stiffened environment. A recent study reported that mechanical tension triggered Piezo1 opening, Ca^2+^ influx, and M2 polarization.^[^ ^84^ ^]^ Our data highlights CaMKK2 as an essential node of this activation pathway in macrophages. The findings using an in vivo wound healing model further validates that the stiff matrices induce pro‐regenerative phenotypic shifts and facilitate vascularization and regeneration, and such mechanical modulation depends on CaMKK2. While *Camkk2* is prominently expressed in macrophages in the skin tissues, other cell types, such as fibroblasts, express considerable levels of *Camkk2*. Using germline *Camkk2* ^−/−^ mice might lead to a phenotypic change in fibroblasts that impacts the healing process. However, it is noteworthy that macrophages and fibroblasts play their roles in distinct time windows during wound healing.^[^ ^61^, ^62^, ^86^ ^]^ Macrophages are the first responders in the acute inflammation stage, and the phenotypic shifts have a profound impact on vascularization and regeneration in later stages. Fibroblasts participate in the proliferative and remodeling stages toward granulation tissue formation and ECM deposition and remodeling. In our in vivo study, we focus on characterizing macrophage's phenotype during the early inflammatory stage of wound healing, when fibroblasts have less impact. Thus, the role of CaMKK2 is investigated only in macrophages at the early stage and yet to be explored in other cell types at later stages during wound healing.

The implications of our findings hold the promise for developing novel therapeutic strategies. Mechanical cues are modulated during various pathological conditions, including cancer, atherosclerosis, and fibrosis.^[^ ^87^, ^88^ ^]^ For example, tumor tissues generally have an increased stiffness than normal tissues, and increased mechanical force signaling can shape the TME by driving the accumulation of pro‐tumoral/immunosuppressive TAM. We have shown that CaMKK2 expressed in macrophages regulates the immunosuppressive TME, inhibiting the tumor growth, and stimulating the anti‐tumor immune response.^[^ ^89^ ^]^ In the current study, we identify CaMKK2 as an important target to modulate the detrimental effects of ECM stiffness on TAM, and possibly other myeloid cell types playing an important role in TME. Countermeasures to block CaMKK2 and suppress stiffness‐induced macrophage polarization would be critical to shaping TME characterized by high stiffness. Noteworthy, our data indicates that CaMKK2 blockers could be leveraged to shape the macrophage landscape in normal, aged, or fibrotic tissues. It is noteworthy that CaMKK2 signaling does not interfere with IL‐4‐induced macrophage polarization. Hence, using CaMKK2 inhibitors would selectively modulate macrophage fate in stiff tissues, whereas the IL‐4‐induced macrophage polarization in non‐tumor tissues would remain unaffected. This is critical because IL‐4‐induced alternative macrophage activation is crucial for its pro‐regenerative and anti‐inflammatory functions in normal tissues.^[^ ^90^, ^91^ ^]^

Downstream proximal components of CaMKK2 signaling in macrophages include AMPK and AKT, which regulate survival metabolic response, and their terminal polarization.^[^ ^68^, ^70^, ^71^ ^]^ We found that CaMKK2 is required to link stiffness signaling with AMPK and AKT phosphorylation. AMPK has been identified as a potent counter‐regulator of inflammatory signaling pathways in macrophages.^[^ ^68^ ^]^ Blocking of AMPK signaling enhances inflammatory cytokines in response to bacterial LPS, while preventing their polarization toward a pro‐regenerative phenotype.^[^ ^65^, ^67^ ^]^ Interestingly, AMPKα can directly bind to and phosphorylate STAT6 at Ser564, amplifying the IL‐4/IL‐13 mediated STAT6 signaling, and further promoting the generation of M2‐like pro‐tumoral macrophages.^[^ ^92^ ^]^ Our data suggests that a similar mechanism could link mechanosensory response to CaMKK2‐AMPK signaling, fostering M2 polarization in response to stiff ECM.

AKT regulates critical function in tumor cells and for this reason, this protein is emerging as a clinically relevant target in cancer therapy.^[^ ^73^ ^]^ Alongside the direct effect on tumor cells, blocking of AKT stimulates T cell functions, promoting the expansion of tumor‐specific lymphocytes.^[^ ^74^ ^]^ Our data indicates that CaMKK2 is required to couple the mechanosensory signaling with AKT activation in macrophages. The effect of Capivasertib on stiffness‐induced M2‐like polarization confirms the biological relevance of CaMKK2‐AKT signaling, suggesting a novel therapeutic application of this drug class to prevent and revert the accumulation of pro‐tumoral TAM in stiff TME, and shaping the macrophage repertoire in other pathological tissues characterized by increased stiffness.

## 4 Conclusion

Our study shows the impact of ECM stiffness on macrophage fate and ECM and identifies a novel mechanism by which matrix stiffening regulates macrophage polarization. We highlight the potential of targeting the stiffness‐sensitive CaMKK2 pathway as a promising strategy to reduce immunosuppression for cancer therapy and regulate the immune response in wound healing. Future studies should extrapolate our findings into other disease models and clinical settings.

## 5 Experimental Section

### Hydrogel Preparation

Methacrylate hyaluronic acid (HA‐MA) was synthesized as previously reported and dissolved in deionized (DI) water at 20 mg mL^−1^.^[^ ^36^ ^]^ Methacrylate type‐I collagen (Col‐MA, Advanced Biomatrix) was dissolved in 20 mm acetic acid at 8 to make a stock solution. Irgacure 2959 (Advanced Biomatrix) was dissolved in methanol at 250 mg mL^−1^. To prepare 1 mL of Col‐MA/HA‐MA solution for soft gel, 500 µL of Col‐MA stock solution (final concentration at 4 mg mL^−1^), 50 µL of HA‐MA stock solution (final concentration at 1 mg mL^−1^), and 410 µL of Dulbecco's Modified Eagle's Medium (DMEM, Gibco) were mixed. The hydrogel solution was neutralized using 40 µL of the neutralization solution (Advanced Biomatrix) and incubated at 37 °C for 30 min for physical crosslinking. To prepare 1 mL of Col‐MA/HA‐MA solution for stiff gel, 750 µL of Col‐MA stock solution (final concentration at 6 mg mL^−1^), 50 µL of HA‐MA stock solution (final concentration at 1 mg mL^−1^), and 140 µL of DMEM were mixed and then neutralized with 60 µL of the neutralization solution and incubated at 37 °C for 30 min. For stiffening, Irgacure 2959 solution (final concentration at 2.5 mg mL^−1^) was added and incubated with the gel for 20 min to allow it to diffuse into the gel. UV light (λ = 365 nm) was applied for 300 s to induce photocrosslinking. The hydrogel was washed with phosphate‐buffered saline (PBS) to remove excessive photocrosslinker.

### Hydrogel Characterization—Storage Modulus Measurement

Bulk stiffness was measured using an HR 20 Rheometer (TA Instruments) equipped with an 8‐mm crosshatched plate. Storage modulus G' was monitored at a fixed strain rate of 1% and fixed frequency of 1 Hz at 37 °C. Axial force was kept at 0.02‐0.03 N during measurements.

### Hydrogel Characterization—Young's Modulus Measurement

Young's modulus was measured using AFM (Nanowizard V BioScience, Bruker) equipped with SAA‐SPH‐10 µm probes (Bruker). Contact mode indentation tests were performed near the center of each hydrogel. The parameters used in this experiment were 5 nN for setpoint, 2 µm for Z length, 2000 Hz for sample rate, and 2 µm ^−1^s for speed. The Young's modulus was calculated by fitting the force‐displacement curves with the Hertz model with an assumed Poisson's ratio of 0.5.

### Hydrogel Characterization—Fiber Analysis

Reflective microscopy (640 nm, Nikon Eclipse Ti2 microscope) was used to analyze collagen‐HA fiber. Fiber diameter and coverage area were analyzed using Nikon NIS software. At least 10 images from 3 replicates were used for quantification.

### Cell Culture—Generation of BMDM

L929 conditioned medium (L929‐CM) was used as a source of macrophage colony‐stimulating factor to generate BMDM.^[^ ^93^ ^]^ To generate L929‐CM, L929 cells were maintained in DMEM supplemented with 10% heat‐inactivated fetal bovine serum (FBS, Hyclone), 2 mM GlutaMax supplement (Thermo Fisher), and 100 U mL^−1^ penicillin‐streptomycin (Thermo Fisher). When the L929 cells reached 70% confluence, the medium was refreshed and then collected after 3–4 days. L929‐CM was then collected, filtered through a 0.22 µm filter, aliquoted, and stored at ‐20 °C until required. The protocol to generate BMDM has been described previously.^[^ ^30^ ^]^ Briefly, bone marrow (BM) cells were collected from the femur and tibiae of WT and *Camkk2^−/−^* male and female C57Bl/6 mice (8‐10 weeks). The BM suspensions were passed through a 70 mm strainer (BD Falcon) and the red blood cells were lysed using RBC lysis buffer (BioLegend). The BM cells were collected and adjusted to 2 × 10^6^ mL^−1^ in DMEM conditional media (10% FBS and 30% L929‐CM). BMs were plated in ultra‐low attachment multi‐well plates (Corning Costar) at 2 × 10^6^/well. On day 4, half of the medium was refreshed and the cells were ready to use.

### Cell Culture—Mammary Tumor Cells

EO771 (parental) and EO771 expressing the infrared fluorescent protein (EO771‐IRFP) cells were provided by Dr. Donald McDonnell, Department of Pharmacology and Cancer Biology, Duke University. Cells were cultured in RPMI‐1640 (Gibco) supplemented with 8% FBS with 4.5g L^−1^ D‐(+)‐glucose solution (Millipore Sigma),10 mM HEPES (Thermo Fisher),1.0 mM sodium pyruvate (Thermo Fisher), and 100 U mL^−1^ penicillin‐streptomycin, as previously described.^[^ ^49^ ^]^ Cells were maintained in a humidified 37 °C CO_2_ incubator and passed at 70% confluence.

### Cell Encapsulation

For cell encapsulation into the hydrogel, the hydrogel solution was prepared and neutralized as described above. Then, BMDM was resuspended in DMEM and mixed with the hydrogel solution at a final concentration of 4 × 10^6^ mL^−1^. Dynamic stiffening was induced 24 h after cell encapsulation by UV crosslinking, as described above. Live/dead staining (Thermo Fisher L3224) and caspase 3/7 (Thermo Fisher C10423) staining were performed to evaluate the survival and apoptosis of macrophages after 48 h of incubation.

For BMDM‐EGFP/EO771‐IRFP co‐culture experiment, WT or *Camkk2* ^−/−^ BMDMs were encapsulated in the hydrogel at a final concentration of 4 × 10^6^ mL^−1^. Hydrogel without BMDM encapsulation was used as the control group. Matrix stiffening was induced using the method described above. EO771 cells were seeded on the hydrogel surface at a density of 2000/gel 24 h after stiffening. After seeding EO771 cells for 48 h, Z‐stack fluorescent images of the hydrogel construct were taken using a Nikon Eclipse Ti2 microscope, and the density of EO771 cells was quantified. The hydrogels were fixed for immunofluorescent staining (see the following section).

### Flow Cytometry—Macrophage Phenotype

After stiffening, the cells were cultured for 48 h. Then, the hydrogel was digested by collagenase IV (400 U mL^−1^, Stem cell technologies) at 37 °C for 1 h. The detached BMDM were collected and stained with PE anti‐CD11b, PE‐Cy7 anti‐F4/80, APC anti‐CD206, and APC‐Cy7 anti‐MHC‐II. Detailed information for all antibodies used was provided in the source file in the supporting information. Counting beads (CountBright, Invitrogen) were added to the analytes to determine the absolute cell number. The stained cells were analyzed using a FACSCanto flow cytometer (BD). CD206/MHC‐II expression was analyzed for CD11b^+^F4/80^+^ gated cells. The percentage of CD206^+^MHC‐II^low^ and CD206^−^MHC‐II^high^ cells was calculated for at least 3 hydrogel replicates.

### Flow Cytometry—CaMKK2 Expression

BMDMs from CaMKK2‐EGFP mice were encapsulated in soft and stiff hydrogels for 48 h. Post‐culture, the cells were harvested, and the expression of the EGFP reporter was quantified using flow cytometry. BMDM generated from regular C57Bl/6 mice were used as negative control for EGFP expression

### Immunofluorescent Staining, Imaging, and Quantification

Hydrogels containing BMDMs or EO771 cells were fixed using 3.7% formaldehyde (Millipore Sigma) for 20 min at room temperature. The constructs were then permeabilized with 1% Triton‐X100 in PBS for 1 h at room temperature and incubated with 3% bovine serum albumin (BSA) in PBS for 1 h at room temperature to reduce the non‐specific binding. The constructs were incubated with primary antibodies, including rabbit anti‐CD206 (1:100, Abcam) and mouse anti‐Ki67 (1:100, Abcam), overnight at 4 °C. After that, the constructs were incubated with corresponding secondary antibodies and phalloidin (Invitrogen). Nuclei were stained with DAPI. The samples were washed 3 times with PBS between any two steps. Fluorescent images were taken by Nikon EclipseTi2 microscope using Z‐stack mode. Maximum intensity projection images were generated by Nikon NIS analysis software. Image quantification was conducted using Nikon NIS analysis software and ImageJ.

### Gene Expression

RNA was extracted using TRIzol reagent (Invitrogen) and purified by RNeasy mini kit (Qiagen). Complimentary DNA was synthesized using GoScript reverse transcription mix (Promega). Gene expression was performed by real‐time RT‐PCR, using Maxima SYBR Green/Fluorescein Master Mix (Thermo Fisher). Primer sequences are provided in the supplementary materials in **Table** **2**. β‐actin (*Actb*) served as the housekeeping gene. The ΔΔCt method was used for data analysis.

**Table 2:** Primers used for real‐time RT‐qPCR.

Gene	Species	Forward [5′‐3′]	Reverse [5′‐3′]
*Actb*	Mouse	GATGTATGAAGGCTTTGGTC	TGTGCACTTTTATTGGTCTC
*Retnla*	Mouse	GATGAAGACTACAACTTGTTCC	AGGGATAGTTAGCTGGATTG
*Chil3*	Mouse	CTTCTAAGACTGGAATGGTG	GTACAAACCTCATAGTAAGCC
*Lgals3*	Mouse	AACACGAAGCAGGACAATAACTGG	GCAGTAGGTGAGCATCGTTGAC
*Irf7*	Mouse	TAAGGTGTACGAACTTAGCC	TACTGCAGAACCTGTGTG
*Tlr7*	Mouse	CTTCAAGAAAGATGTCCTTGG	AAATTTGTCTCTTCCGTGTC
*Ifit3*	Mouse	TTCTGCTGCCTGGGCTTCATAG	ACCAAGGTGCTGATGTTCAGGC

### RNA‐Sequencing and Differential Gene Expression Analysis

RNA was extracted and purified as described above. RNA from 2 biological replicates for each experimental point was used for RNA sequencing (Novogene). Messenger RNA was purified from total RNA using poly‐T oligo‐attached magnetic beads. After fragmentation, the first strand of cDNA was synthesized using random hexamer primers. The second strand cDNA was synthesized using dUTP, instead of dTTP. The directional library was ready after end repair, A‐tailing, adapter ligation, size selection, USER enzyme digestion, amplification, and purification. PolyA‐selected, strand‐specific libraries were prepared and sequenced on the Illumina Novaseq Platform, which generates twenty million PE150 reads output data (6GB). The original raw data from the Illumina platform were transformed into Sequenced Reads, known as Raw Data or RAW Reads, by base calling. Raw data were recorded in a FASTQ file, which contains sequencing reads and corresponding sequencing quality.^[^ ^94^ ^]^ The RNAseq dataset was analyzed by Dr. Jianhong Ou, Regeneromics, Duke Regeneration Center, Duke University. Briefly, the RNA‐seq datasets were processed using the RNA‐seq pipeline implemented in snakePipe.^[^ ^95^ ^]^ Specifically, paired‐end reads were mapped to the mouse genome (mm10) using STAR aligner^[^ ^96^ ^]^ with default settings. Reads mapped to genes were counted using featureCounts^[^ ^97^ ^]^ to obtain the gene‐level counts, and differential expression was done using DESeq in R.^[^ ^98^ ^]^ The complete list of identified genes was used to generate volcano plots. iDEP‐integrated web platform (http://ge‐lab.org/idep/) was used for volcano plots, Venn diagrams, and enrichment pathway analysis.^[^ ^99^ ^]^ STRING database (https://string‐db.org/)^[^ ^100^ ^]^ was used for network analysis and representation. Raw data are accessible through NCBI Gene Expression Omnibus, accession number GSE273834.

### Animal Study

Division of Laboratory Animal Resources in Duke University Medical Center engineered genotype and breed. All animal care and experiment procedures were conducted according to the National Institutes of Health guidelines. The animal protocols were approved by the Division of Laboratory Animal Resources of Duke University (Protocol # A207‐23‐10 and A062‐23‐03 for tumor and wound healing studies, respectively).

### Tail‐Vein Metastasis Studies

WT and germline *Camkk2^−/−^* mice have been previously described.^[^ ^101^ ^]^ EO771‐Luc cells expressing a bioluminescent trackable marker (fireflight luciferase) were acquired from the laboratory of Donald McDonnell (Duke University, Durham, NC), and have been described previously.^[^ ^102^ ^]^ EO771 (EO771‐Luc) cells were inoculated in the tail vein of 8–10 weeks WT and *Camkk2^−/−^* mice, and tumor growth was monitored as previously reported.^[^ ^102^ ^]^ Briefly, 300000 tumor cells were injected into the lateral tail vein. To monitor tumor growth, mice were regularly anesthetized with isoflurane and intravenously administered 100 µL luciferin (Regis Technologies, cat no. 1–360243–200) at the standard concentration of 50 mM. Bioluminescence was then measured using an IVIS (in vivo imaging system) Lumina XR optical imaging system with an exposure time of 2 to 3 min. Mice were euthanized at the humane or experimental endpoint (60 days after tumor cell tail vein injection), whichever came first. Lungs were removed to assess the presence of tumor mass.

### Wound Healing Model

Male 12‐week‐old C57BL/6J (WT) or *Camkk2^−/−^* mice were anesthetized using isoflurane. After hair removal, a biopsy punch (AD surgical) was used to create two full‐thickness, 5‐mm‐diameter wounds on the dorsal skin. Silicone wound splints (Grace Bio‐Labs) were applied and sutured onto the skin to reduce the contraction of excisional wounds. Soft and stiff hydrogels were applied topically to the wound and covered by Tegaderm film (3M). On days 3 and7, the animals were euthanized, and the wound tissue was extracted for flowhistology and immunohistochemical analyses.

### Histology and Immunohistochemical Staining (IHC)—Lung Tumor

Lung sections were stained with 3 antibodies to detect myeloid cells, tumor epithelial cells, and proliferating cells using rabbit polyclonal CD68 antibody (Abcam, catalog#: ab125212), rabbit monoclonal antibody EGFR (D1P9C; Cell Signaling catalog#: 71655s), and rabbit monoclonal antibody Ki67 (SP6; Abcam catalog#: ab16667), respectively. Ki67 and EGFR were diluted at 1:200, while CD68 was at 1:50 dilution, with Discovery Ab Diluent (cat# 760‐108). IHC tests were performed using the Ultra Discovery automated staining platform. The tissue sections were pretreated (epitope retrieval) with Roche cell conditioning solution CC1 (Roche catalog#. 950‐124) for 32 min, then incubated first with Ki67 for 20 min at 37 °C. After binding of the primary antibody, Roche anti‐rabbit HQ (cat.760‐4815) was applied and incubated for 8 min, followed by 8 min incubation with anti‐HQ HRP (760‐4820) for antigen detection. For visualization, Disc. ChromoMap DAB (760‐159) was applied and incubated for 5 min. Before the dual sequence EGFR was applied, the first sequence reaction was denatured by raising the temperature to 93 °C for 8 min. Then Disc inhibitor (760–4840) was added to neutralize the HRP activity in the first sequence. Rabbit monoclonal EGFR was applied and incubated for 20 min at 37 °C. After binding the primary antibody, Roche anti‐rabbit HQ was applied and incubated for 8 min, followed by 8 min of incubation with anti‐HQ HRP. For visualization, Discovery Purple HRP (760‐229) was applied and incubated for 16 min. Before the triple sequence CD68 was applied, the denaturing and the neutralization steps, as mentioned above, were repeated. CD68 was added and incubated for 32 min at 37 °C. The binding of the CD68 was detected with Roche anti‐rabbit‐NP (760‐4817) for 12 min, followed by incubation with anti‐NP AP (760‐4827) for another 12 min. For visualization, Discovery Yellow (760‐239) was applied and incubated for 20 min. The nucleus was counterstained for 4 min with hematoxylin (760‐2021) and incubated for 4 min with bluing reagent (760‐2037). Same species negative control antibodies were run in parallel with each primary antibody. Histological sections were digitized via an Aperio GT450 whole slide scanner at 40x (Leica Biosystems, Buffalo Grove IL). Images were visualized utilizing the Aperio ImageScope software V12.3.3.5048 (Leica Biosystems, Buffalo Grove IL). QuPath‐0.5.1‐x64 was used for quantitative analysis of images.

### Histology and Immunohistochemical Staining (IHC)—Wound Healing Model

After the euthanasia of the animal, the hydrogel and surrounding wound tissue were collected and fixed in 4% paraformaldehyde overnight, embedded in paraffin, and serially sectioned at 5 µm. Hematoxylin and eosin staining was performed. For immunohistochemical analysis, following deparaffinization, antigen retrieval, and blocking, the sections were incubated with primary antibodies, including rat anti‐F4/80 (1:100, eBioscience), rabbit anti‐CD206 (1:100, Abcam), rabbit anti‐CD86 (1:100, Invitrogen), rabbit anti‐alpha smooth muscle actin (1:100, Abcam) and rat anti‐CD31 (1:150, eBioscience), overnight at 4 °C. Detailed information for all antibodies used was provided in the source file in the supporting information. The sections were washed 3 times in PBS and incubated with corresponding secondary antibodies. Nuclei were stained with DAPI. Quantification of the staining was performed using Nikon NIS analysis software and ImageJ.

### Western Blot

At pre‐determined time points, the hydrogel was digested by collagenase IV (400 U mL^−1^) at 37 °C for 1 h. The detached BMDM were lysed using radioimmunoprecipitation assay buffer (Thermo Fisher) containing protease and phosphatase inhibitor (Thermo Fisher). Protein concentration was determined by a BCA assay kit (Thermo Fisher). Protein was boiled at 95 °C for 5 min, loaded at a concentration of 20 µg per lane, and separated by sodium dodecyl sulfate–polyacrylamide gel electrophoresis. Low‐fluorescence polyvinylidene fluoride membranes (Bio‐rad) were used to transfer the proteins at 4 °C overnight. Protein transfer was confirmed using Ponceau‐S staining. The blots were blocked for 1 h with 5% BSA in Tris‐buffered saline containing 0.1% Tween 20 (TBST) for phosphorylated protein detection and 5% milk in TBST for non‐phosphorylated protein detection. Then the membranes were incubated with primary antibodies at 4 °C. The primary antibodies used in this study include GAPDH, phosphor‐AKT, AKT, phosphor‐AMPK, AMPK, phosphor‐STAT6, and STAT6. Detailed information for all antibodies used was provided in the source file in the supporting information. After overnight incubation, the blots were washed with TBST three times and incubated with anti‐mouse or rabbit HRP‐conjugated secondary antibodies. Immunoblots were detected by an enhanced chemiluminescence western blotting substrate (Thermo Fisher) and imaged using ChemiDoc Imaging System (Bio‐Rad). Analyses were performed using ImageJ. The ratio of the expression of phosphorylated protein to total protein was quantified and normalized to the WT soft group. Original blots were included in Figure S7C (Supporting Information).

### Calcium Assay

Calcium ion concentration was measured in soft and stiff hydrogels using a calcium assay kit (Abcam 102505). Hydrogels without cells were fabricated and incubated in BMDM culture medium. After 48 h, hydrogel and media (supernatant) were separated and the calcium ion level was determined using the protocol provided by the manufacturer. Hydrogel was washed with calcium‐free DPBS before testing to remove the calcium in the serum.

### Statistical Analysis

All experiments were performed at least in biological triplicates (N = 3 or more). All data were presented as means ± SD, as indicated in the figure. Statistical analysis was performed between 2 groups using t‐tests, or among 3 or more groups using one‐way analysis of variance (ANOVA) with Tukey's post hoc test, or as indicated in the figure legend, using GraphPad Prism 10. Significance levels were set at *p < 0.05, **p < 0.01, ***p < 0.001, and ****p<0.0001.

## Conflict of Interest

The authors declare no conflict of interest.

## Author Contributions

L.R and S.G are also co‐senior authors. S.G., L.R., and Y.G. conceived and designed the study; Y.G., M.Z., J.S., M.N., T.A., and R.K. carried out the experiments; S.G., L.R., and Y.G. contributed to funding acquisition; S.G. and L.R. supervised the project; Y.G., L.R., and S.G. wrote the original draft, reviewed, and edited.

## Supporting information



Supporting Information

## Data Availability

This article and its supplementary data file include all data generated or analyzed in this study. Raw data points are available from the corresponding author on a reasonable request. RNA‐seq data are accessible through NCBI Gene Expression Omnibus, accession number GSE273834. This paper does not report the original code.
