# Supplementary material for: CaMKK2 Regulates Macrophage Polarization Induced by Matrix Stiffness: Implications for Shaping the Immune Response in Stiffened Tissues
Source: Adv Sci (Weinh). 2025 Mar 4;12(16):2417778. doi: 10.1002/advs.202417778 (PMC12021110; doi:10.1002/advs.202417778)
Supplement: Supplementary file 1 — Supporting Information [file ADVS-12-2417778-s001.docx]

Supporting Information

CaMKK2 regulates macrophage polarization induced by matrix stiffness: implications for shaping the immune response in stiffened tissues

Ya Guan, Min Zhang, Jiyeon Song, Marcos Negrete, Tyler Adcock, Reeva Kandel, Luigi Racioppi* and Sharon Gerecht*


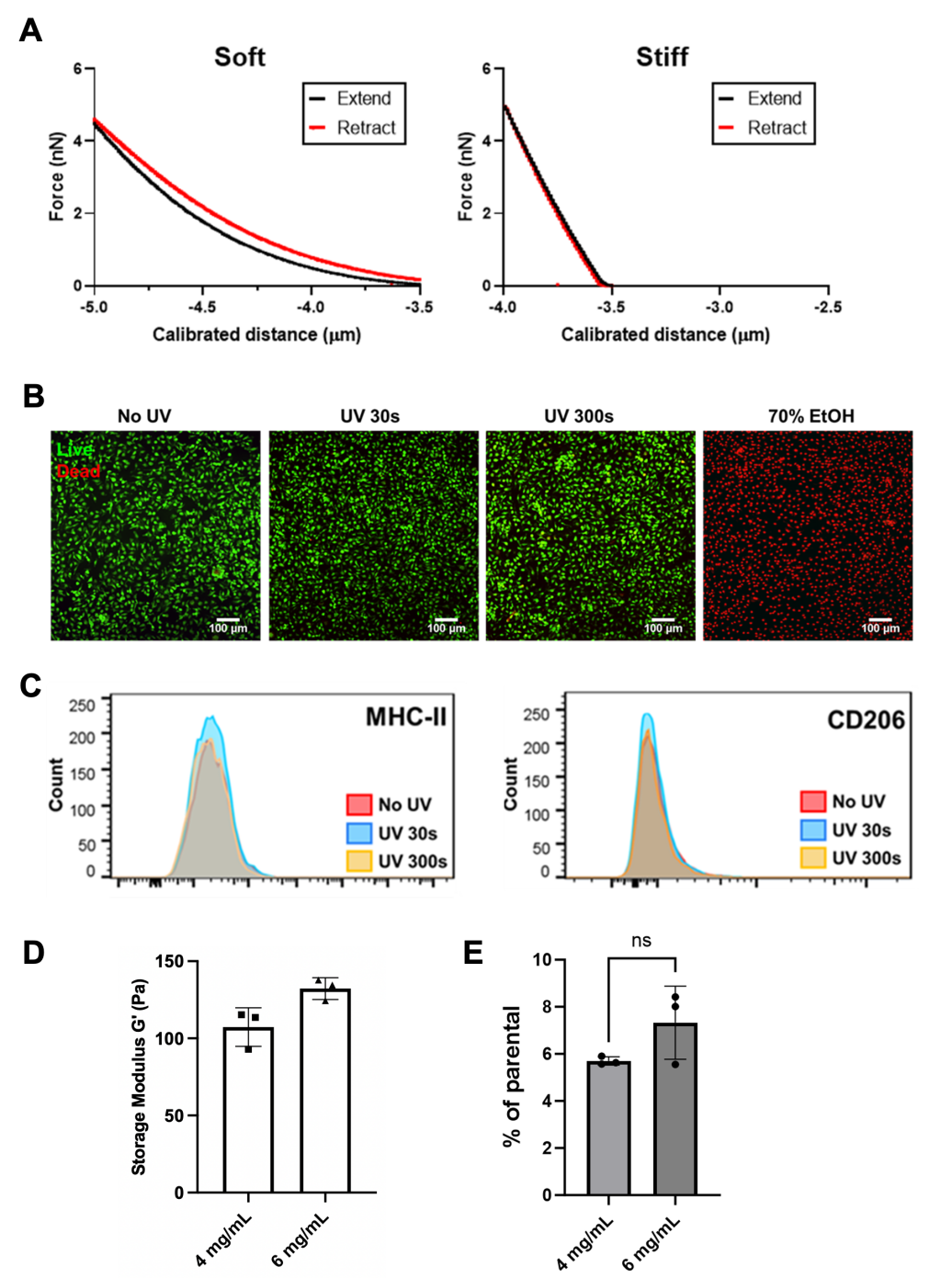


**Figure S1. Fabrication and characterizations of the dynamically stiffening hydrogel.**

(A) Force distance curve of the AFM measurement used to determine the Young’s modulus of soft and stiff Col-HA hydrogels. (B) Representative live/dead staining images of BMDMs treated with or without UV and cultured for 24 hours. (C) MHC-II and CD206 expression on CD11b^+^F4/80^+^ gated BMDMs treated with or without UV and cultured for 24 hours. (D) Rheology measurement of the storage modulus of the non-crosslinked hydrogels with different collagen concentrations. (N=3, p=0.22). (E) CD206^+^MHC-II^low^ cell percentage of CD11b^+^F4/80^+^ gated BMDMs in non-crosslinked hydrogels with different collagen concentrations.


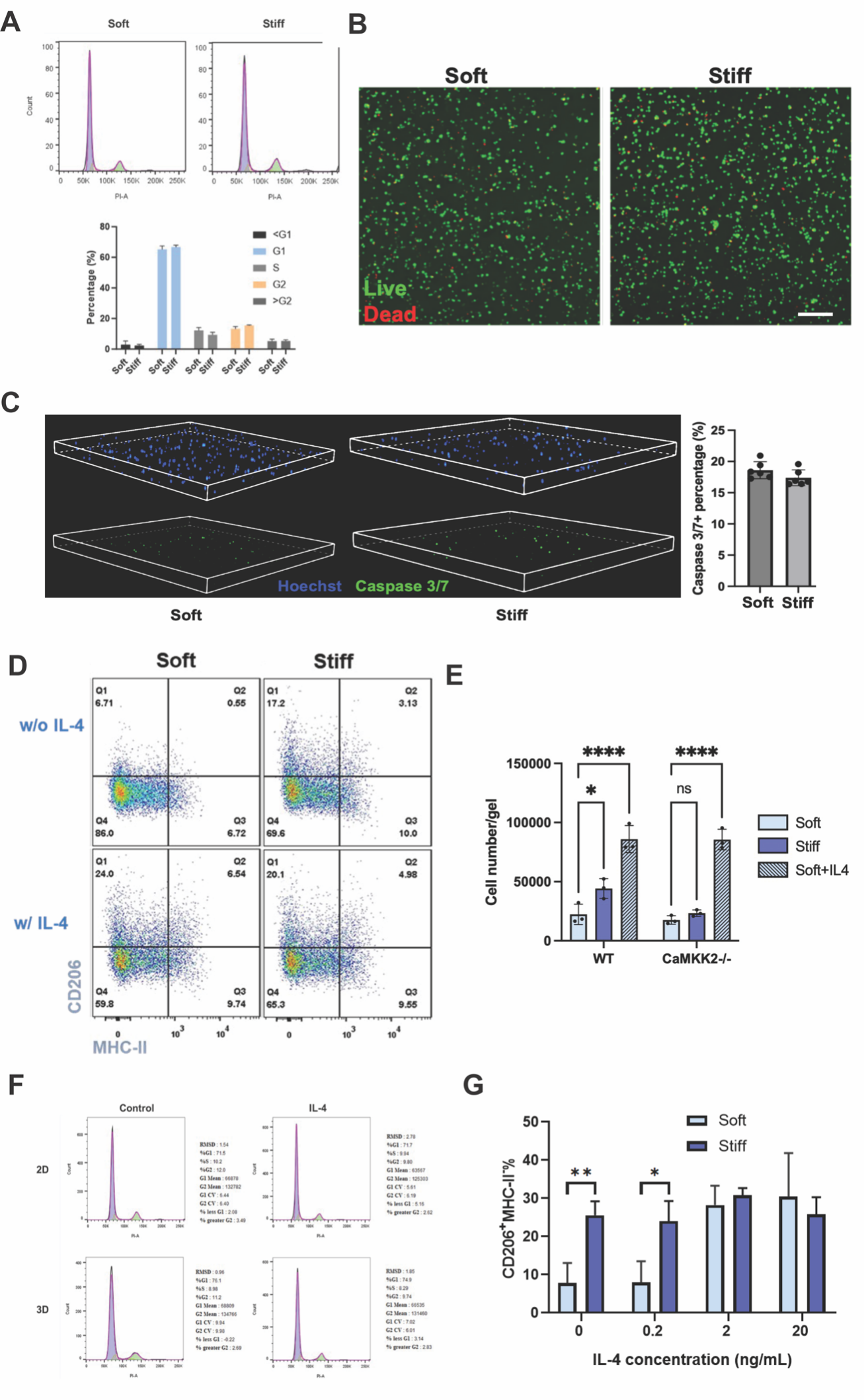


**Figure S2. BMDM’s phenotypic polarization upon dynamic stiffening.**

(A) Flow cytometry histograms of propidium iodide (PI) staining of the BMDMs to analyze the cell cycle in soft and stiff hydrogels. (B) Live and dead staining of BMDMs encapsulated in soft and stiff hydrogels after 2 days. Scale bar = 50 µm. (C) Caspase 3/7 staining and quantification to evaluate macrophage apoptosis in soft and stiff hydrogels after 2 days. N =3.

(D) Dot plot flow cytometry analysis of CD206 and MHC-II expression on CD11b^+^F4/80^+^ gated BMDMs encapsulated in the hydrogels after 2 days. (E) CD206^+^MHC-II^low^ cell number count from CD11b^+^/F4/80^+^ gated BMDMs encapsulated in the hydrogels after 2 days, N = 3 (Two-way ANOVA, *p<0.05, ****p<0.0001). (F) Flow cytometry histograms of propidium iodide (PI) staining of the BMDMs to analyze the cell cycle with or without IL-4 (20 ng/mL). (G) Flow cytometry analysis of CD206/MHC-II expression on CD11b^+^F4/80^+^ gated BMDMs encapsulated in the hydrogels and treated with different concentrations of IL-4 after 2 days.


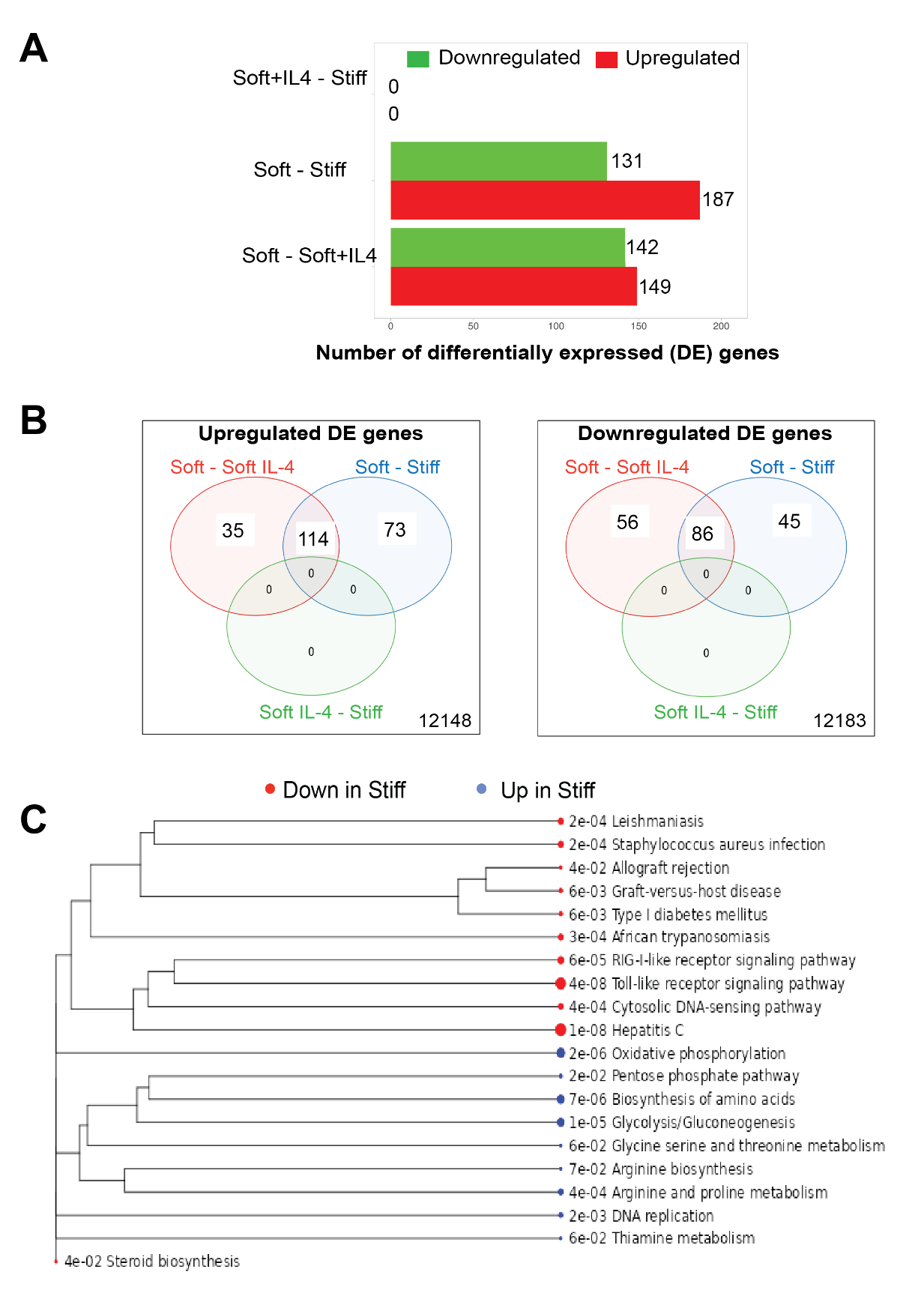


**Figure S3. Transcriptomics analysis of the BMDMs in dynamically stiffened hydrogel.**

(A) Number of differentially expressed genes in soft, soft+IL-4, and stiff conditions. (B) Venn diagram of the differentially expressed genes. (C) Gene ontology pathway analysis based on differentially expressed genes comparing soft and stiff conditions.


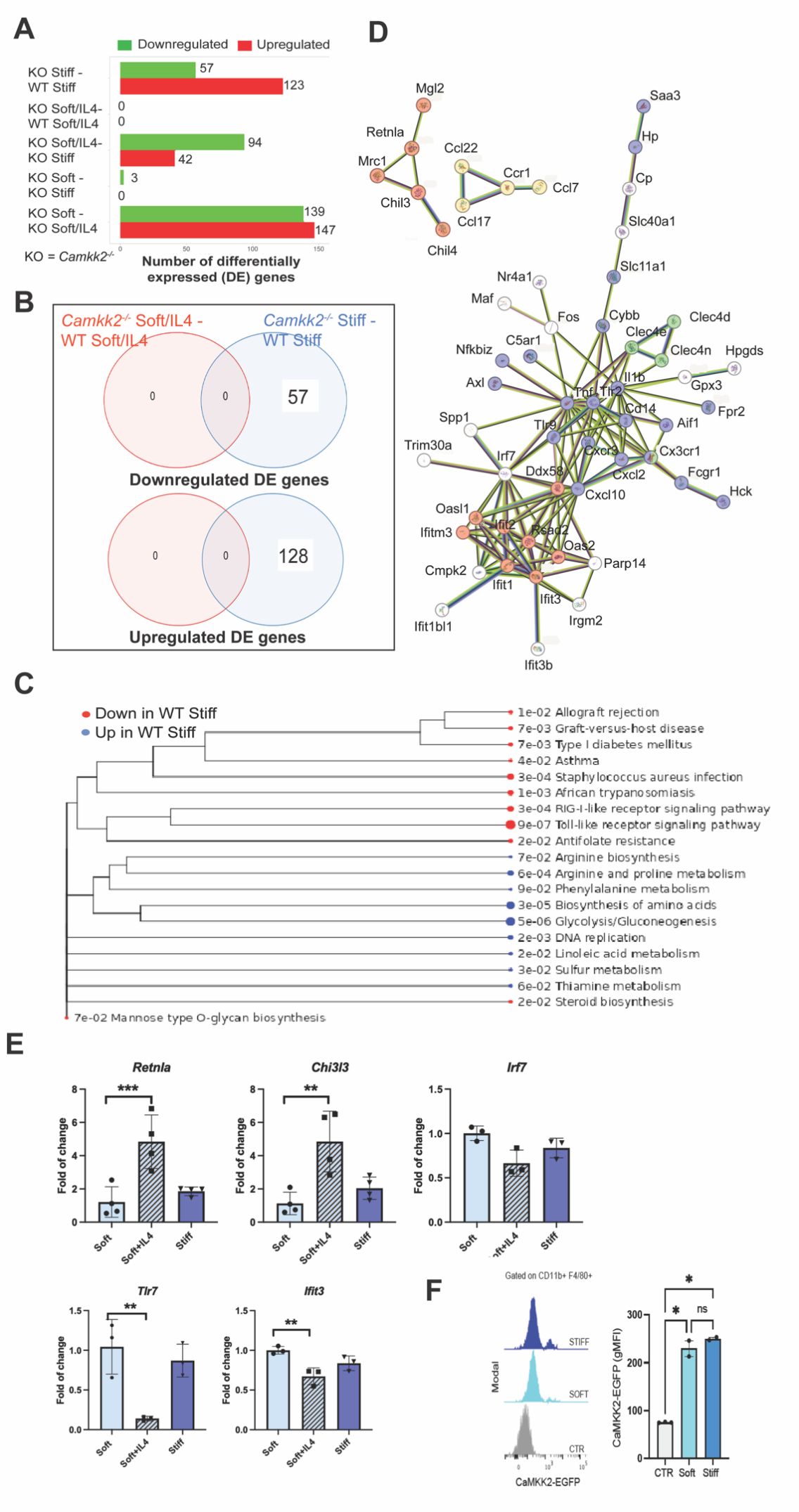


**Figure S4. Role of Camkk2 in stiffness-induced BMDM polarization.** (A) The number of differentially expressed genes in WT and *Camkk2^-/-^* (KO) BMDMs in soft, soft+IL-4, and stiff conditions. (B) Venn diagram of the differentially expressed genes. (C) Gene ontology pathway analysis based on differentially expressed genes comparing WT stiff and *Camkk2^-/-^* stiff conditions. (D) Network analysis of DE genes upregulated (top) and downregulated (bottom) in WT stiff compared to *Camkk2^-/-^* BMDM. (E) Real-time RT-qPCR analysis of pro-regenerative and pro-inflammatory genes expressed by *Camkk2^-/-^* BMDMs in soft, soft+IL-4, and stiff conditions. (F) Effects of dynamic stiffening on Camkk2 promoter activity in BMDM. Macrophages were generated from the bone marrow of CaMKK2-EGFP reporter mice and encapsulated in soft and stiff hydrogels. After 48 hours, BMDM were recovered from hydrogels and EGFP expression was assessed by flow cytometry. (Left) EGFP expression on CD11b^+^/F4/80^+^ BMBM recovered from stiff and soft hydrogels. BMDM generated from regular C57Bl/6 mice were used as negative control for EGFP expression. (Right) EGFP geometric mean (gMFI). Two-way ANOVA, *p<0.05.

**
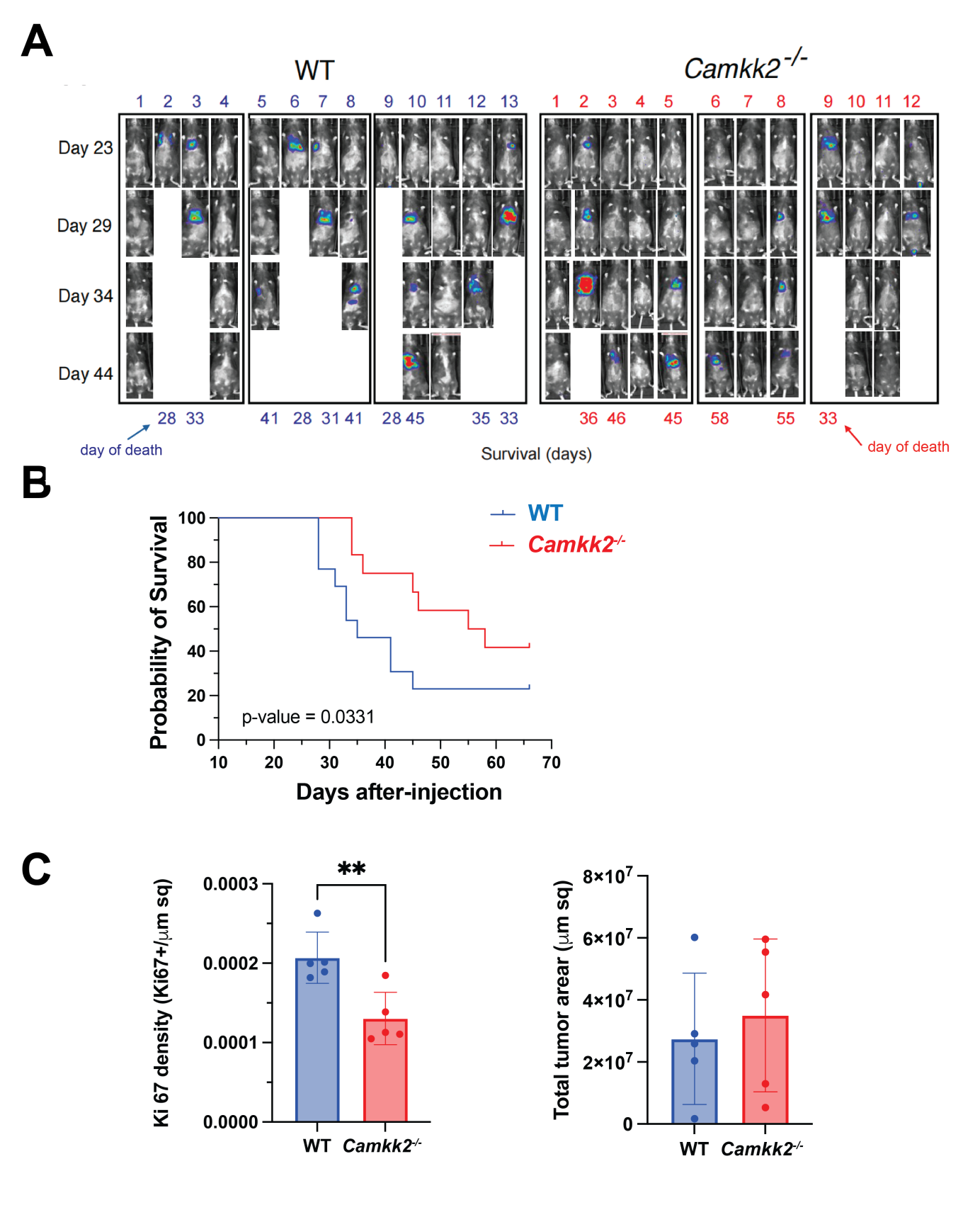
**

**Figure S5. Deletion of *Camkk2* in the host restrains the proliferation of mammary tumor cells.** EO771-Luc cells (200,000 cells/mouse) were inoculated in the tail vein of WT and *Camkk2^-/-^* mice. (A) The whole-body metastatic burden of EO771-Luc cells was measured using luciferin bioluminescence imaging. (B) Survival analysis. The p-value was calculated using Gehan-Breslow-Wilcoxon. (C) EO771-Luc cells were injected in the tail vein of WT and *Camkk2^-/-^* mice and mice were euthanized 30 days after tumor cell injection. Lungs infiltrated by tumors of comparable size were collected from 5 WT and 5 *Camkk2*^-/-^ mice, and multiplex stained with CD68, EGFR, and Ki67 antibodies. Nuclei were identified by hematoxylin staining. The density of Ki67⁺ cells and the total area of tumor nodules in each lung were assessed using QuPath software (left and right, respectively). At least 2 nodules for each tumor were analyzed. T-tests, p** < 0.01.


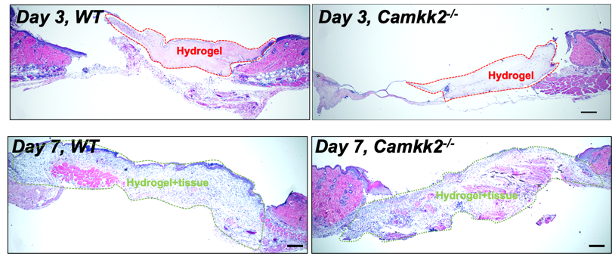


**Figure S6**. **Histological assessment of wounds treated with Col-HA gel.** Low magnification H&E images of the wounds on days 3 (A) and 7 (B). Scale bar = 200 µm.

**
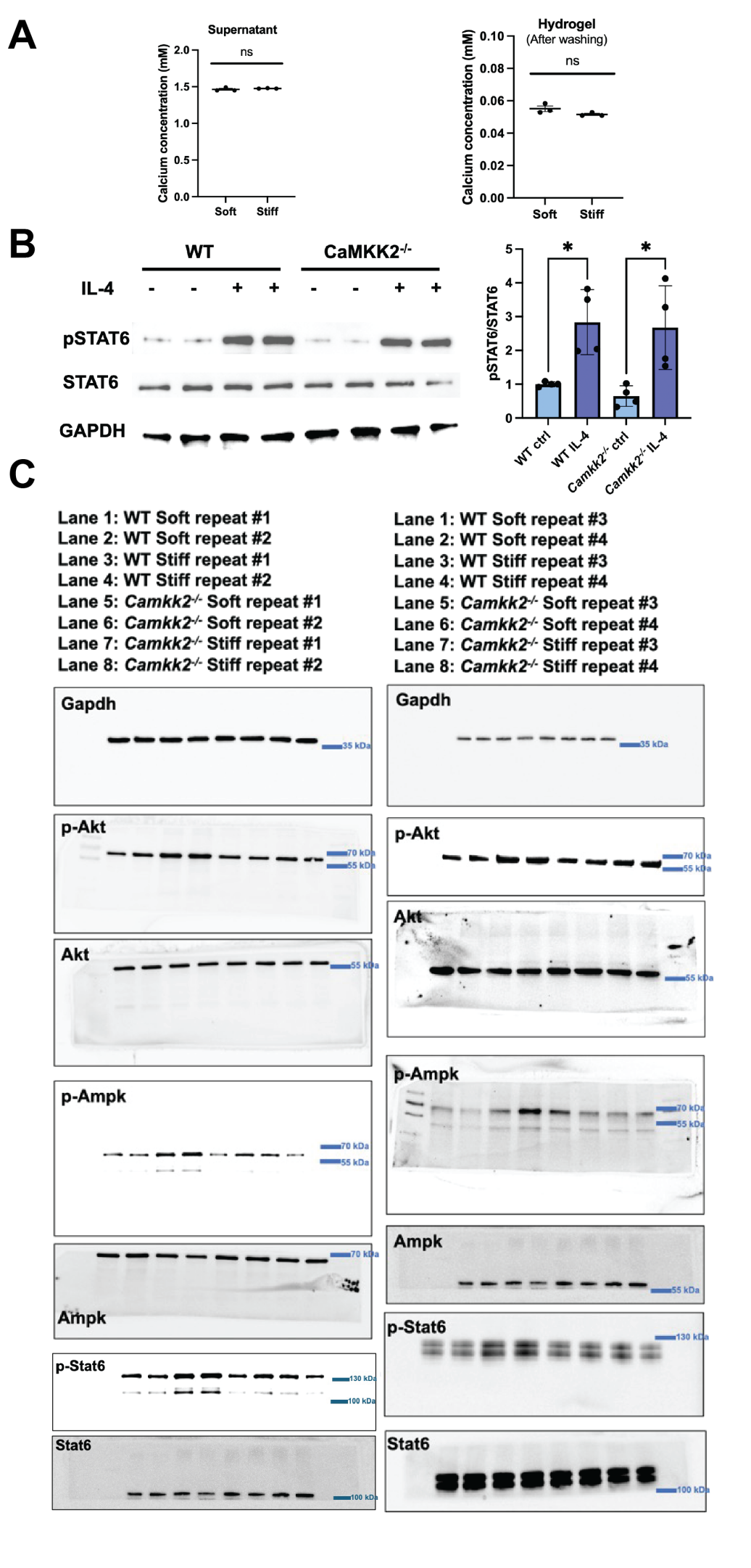
**

**Figure S7. Mechanistic study of stiffness-induced CaMKK2-dependent BMDM polarization.**  (A) Calcium ion concentration measurement in the media (supernatant) and hydrogels with or without stiffening. N = 3. (One-way ANOVA) (B) Western blot to analyze (phosphorylated) Stat6 expressed in WT or *Camkk2*^-/-^ BMDMs encapsulated in soft hydrogel and treated with or without IL-4. Gapdh was used as a protein loading control. The ratio of the expression of phosphorylated Stat6 to total Stat6 was quantified and normalized to the WT control group. N = 4. (One-way ANOVA, *p < 0.05). (C) Original Western blot images.
